# High-stability spherical lanthanide nanoclusters for magnetic resonance imaging

**DOI:** 10.1093/nsr/nwad036

**Published:** 2023-02-16

**Authors:** Hai-Ling Wang, Donglin Liu, Jian-Hua Jia, Jun-Liang Liu, Ze-Yu Ruan, Wei Deng, Shiping Yang, Si-Guo Wu, Ming-Liang Tong

**Affiliations:** Key Laboratory of Bioinorganic and Synthetic Chemistry of Ministry of Education, School of Chemistry, Sun Yat-sen University, Guangzhou 510006, China; College of Chemistry and Materials Science, Shanghai Normal University, Shanghai 200234, China; Key Laboratory of Bioinorganic and Synthetic Chemistry of Ministry of Education, School of Chemistry, Sun Yat-sen University, Guangzhou 510006, China; Key Laboratory of Bioinorganic and Synthetic Chemistry of Ministry of Education, School of Chemistry, Sun Yat-sen University, Guangzhou 510006, China; Key Laboratory of Bioinorganic and Synthetic Chemistry of Ministry of Education, School of Chemistry, Sun Yat-sen University, Guangzhou 510006, China; Key Laboratory of Bioinorganic and Synthetic Chemistry of Ministry of Education, School of Chemistry, Sun Yat-sen University, Guangzhou 510006, China; College of Chemistry and Materials Science, Shanghai Normal University, Shanghai 200234, China; Key Laboratory of Bioinorganic and Synthetic Chemistry of Ministry of Education, School of Chemistry, Sun Yat-sen University, Guangzhou 510006, China; Key Laboratory of Bioinorganic and Synthetic Chemistry of Ministry of Education, School of Chemistry, Sun Yat-sen University, Guangzhou 510006, China

**Keywords:** *T_1_*-weighted MRI, lanthanide clusters, high stability, low toxicity, assembly mechanism

## Abstract

High-nuclear lanthanide clusters have shown great potential for the administration of high-dose mononuclear gadolinium chelates in magnetic resonance imaging (MRI). The development of high-nuclear lanthanide clusters with excellent solubility and high stability in water or solution has been challenging and is very important for expanding the performance of MRI. We used *N*-methylbenzimidazole-2-methanol (HL) and LnCl_3_·6H_2_O to synthesize two spherical lanthanide clusters, Ln_32_ (Ln = Ho, Ho_32_; and Ln = Gd, Gd_32_), which are highly stable in solution. The 24 ligands L^−^ are all distributed on the periphery of Ln_32_ and tightly wrap the cluster core, ensuring that the cluster is stable. Notably, Ho_32_ can remain highly stable when bombarded with different ion source energies in HRESI-MS or immersed in an aqueous solution of different pH values for 24 h. The possible formation mechanism of Ho_32_ was proposed to be Ho(III), (L)^−^ and H_2_O → Ho_3_(L)_3_/Ho_3_(L)_4_ → Ho_4_(L)_4_/Ho_4_(L)_5_ → Ho_6_(L)_6_/Ho_6_(L)_7_ → Ho_16_(L)_19_ → Ho_28_(L)_15_ → Ho_32_(L)_24_/Ho_32_(L)_21_/Ho_32_(L)_23_. To the best of our knowledge, this is the first study of the assembly mechanism of spherical high-nuclear lanthanide clusters. Spherical cluster Gd_32_, a form of highly aggregated Gd(III), exhibits a high longitudinal relaxation rate (1 T, *r*_1_ = 265.87 mM^−1^·s^−1^). More notably, compared with the clinically used commercial material Gd-DTPA, Gd_32_ has a clearer and higher-contrast *T*_1_-weighted MRI effect in mice bearing 4T1 tumors. This is the first time that high-nuclear lanthanide clusters with high water stability have been utilized for MRI. High-nuclear Gd clusters containing highly aggregated Gd(III) at the molecular level have higher imaging contrast than traditional Gd chelates; thus, using large doses of traditional gadolinium contrast agents can be avoided.

## Teaser

Spherical cluster Gd_32_, which has highly aggregated Gd(III), exhibits a high longitudinal relaxation rate, and excellent *T*1-weighted MRI imaging effect *in vivo* and *in vitro*.

## INTRODUCTION

In magnetic resonance imaging (MRI) or spin imaging, the electromagnetic waves generated by energy attenuation differences in different structural environments inside tissue are monitored by an external gradient magnetic field to form an image of the structure inside the tissue [1,2]. Because complex lesions, such as tumors, can be difficult to find and clearly visualize, it is usually necessary to use contrast agents (CAs) to enhance the MRI signal and improve diagnostic capability. The CAs indirectly change the signal intensity of the tissue through internal and external relaxation effects and the magnetic susceptibility effect to increase the difference in pixel intensity between the diseased tissue and the normal tissue [3–5]. Discrete mononuclear Gd(III) complexes (Gd-DTPA, Gd-DOTA, *etc.*) formed by Gd(III), which has a large magnetic moment and a long electron relaxation time, and poly(aminocarboxylate) chelators have been used as clinical MRI CAs [6]. These Gd(III) complexes mainly enhance tissue contrast in MRI by influencing and regulating the relaxation time of internal and external water protons [7–9]. Due to the low content of Gd(III), the CAs currently used in clinical practice usually require high doses to achieve effective contrast and resolution. However, the use of high-dose Gd(III)-based CAs has high toxicity that may induce many diseases, such as nephrogenic systemic fibrosis [1]. Effective solutions can be proposed based on the Solomon–Bloembergen–Morgan (SBM) paramagnetic relaxation theory: multiple single-nuclear or low-nuclear gadolinium complexes are connected through multicomponent integration to obtain CAs with high relaxation rate, high resolution and high contrast [10–12]. Although some progress has been made, the process is still limited to porous systems such as coordination molecular cages and metal-organic frameworks [13,14]. These systems have limited solubility, and it is difficult to keep them stabilized in body fluids for a long time. In addition, in recent years, iron-based and manganese-based complexes have been developed as CAs to replace gadolinium-based chelates, but their relaxation rate still needs to be improved, and the *in vivo* toxicity of these complexes is not clear [15–18]. Coordination-driven self-assembly provides an efficient method for the development of polynuclear Ln-assemblies with rigid structures, high molecular weight, intermediate sizes, and good solubilities, which all facilitate high relaxivity [13,14]. High-nuclear gadolinium clusters with superior solubility and biocompatibility can highly aggregate Gd(III) at the molecular level, which has attractive potential for MRI CAs [13]. Therefore, it is both challenging and important to develop high-nuclear gadolinium cluster aggregates to have water solubility and water stability for MRI uses.

The design and synthesis of high-nuclear lanthanide clusters with specific connections, attractive structures and rich functions have always received extensive attention [19]. To date, many different shapes and types of high-nuclear lanthanide clusters have been constructed by ligand hydrolysis and template methods [20–25], such as cage Gd_140_ [20], hamburger Dy_76_ [21], and tubular Dy_72_ [22], and they have been applied to single-molecule magnets, magnetocaloric effects and proton conduction fields [26–28]. Although considerable progress has been made, high-nuclear lanthanide clusters are still largely limited to the solid-state [29,30]. Although these solid-state properties are important, the solubility and stability of these clusters in solution are also important factors for the development of other functions, such as bioimaging, therapy and catalysis [31,32]. However, it is not easy to design and synthesize high-nuclear lanthanide clusters with high solubility and stability in solution [33]. Choosing appropriate ligands, wrapping the cluster cores during the self-assembly process and forming a protective effect opens up a new way for the design and synthesis of high-nuclear lanthanide clusters with high stability in solution.

**Scheme 1.**
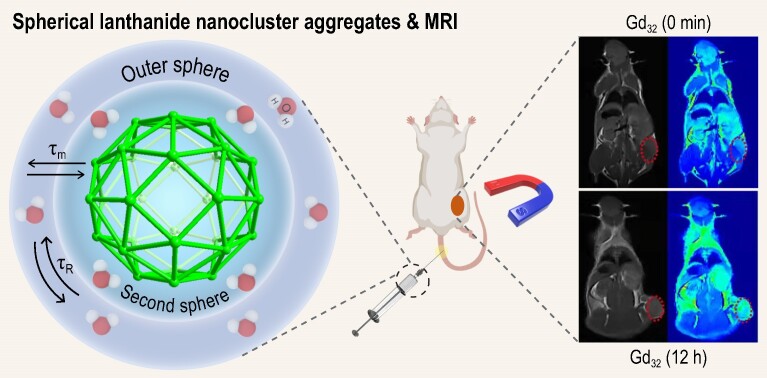
Spherical nanocluster Gd_32_ acts as a *T*_1_-weighted MRI CA for the diagnosis of tumors.

Herein, we reacted *N*-methylbenzimidazole-2-methanol (HL) with LnCl_3_·6H_2_O under solvothermal conditions to obtain two spherical lanthanide nanoclusters, Ln_32_ (Ln = Ho, Ho_32_; and Ln = Gd, Gd_32_). In the Ln_32_ structure, the metal centers Ln(III) are all on the spherical surface and are connected with 6 *μ*_4_-O^2^^−^ and 48 *μ*_3_-OH^−^, and the ligands L^−^ are located on the outside of the sphere and tightly wrap the cluster core, thereby ensuring the stability of the cluster. HRESI-MS and PXRD jointly verified the stability of spherical cluster Ln_32_ in organic solvents and aqueous solutions. The assembly mechanism of Ho_32_ is proposed to be Ho(III), (L)^−^ and H_2_O → Ho_3_(L)_3_/Ho_3_(L)_4_ → Ho_4_(L)_4_/Ho_4_(L)_5_ → Ho_6_(L)_6_/Ho_6_(L)_7_ → Ho_16_(L)_19_ → Ho_28_(L)_15_ → Ho_32_(L)_24_/Ho_32_(L)_21_/Ho_32_(L)_23_. Cluster Gd_32_, which has a high longitudinal relaxation rate and low cytotoxicity, exhibits better MRI imaging contrast than Gd-DTPA at both the solution and cell levels (Scheme 1). The same doses (100 μL) of Gd_32_ and Gd-DTPA containing the same Gd(III) ion concentration (0.5 mM) were injected through the tail vein into BALB/c mice carrying the 4T1 tumor model. Notably, compared with Gd-DTPA, Gd_32_ results in clearer MRI imaging contrasts and has a greater ability to mark tumors (Scheme 1). In addition, Gd_32_ can be cleared from the body in a short time through the kidneys and liver. To the best of our knowledge, this is the first development of high-nuclear gadolinium nanoclusters with highly aggregated Gd(III) as MRI CAs, which effectively avoids the use of high-dose low-nuclear gadolinium chelates.

## RESULTS AND DISCUSSION

### Synthesis and structure analysis of Ln_32_ clusters (Ln = Ho and Gd)

**Figure 1.**
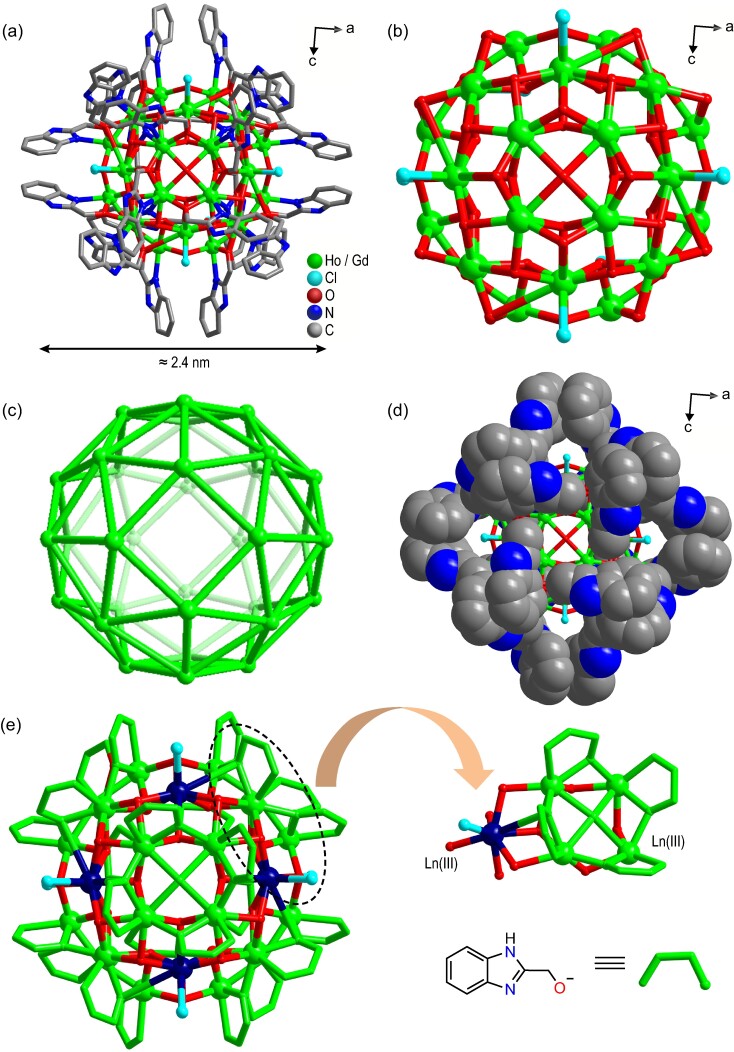
Molecular structure (a), core structure (b) and metal connection (c) of Ho_32_ (free Cl^−^ ions and solvent molecules have been omitted for clarity). (d) Space-filling mode of Ho_32_, in which all ligands protected the cluster core. (e) Simplified molecular structure diagram of Ho_32_, which contains Ho(III) ions in two different coordination environments.

The ligands HL and HoCl_3_·6H_2_O were allowed to react for 48 h in a closed reaction vessel under solvothermal conditions at 100°C and then placed in an open glass bottle at room temperature to volatilize for 12 h. Then, block orange crystals of Ho_32_ were obtained (Fig. S1). Single crystal X-ray diffraction (SCXRD) structure analysis shows that Ho_32_ crystallizes in the *C*2/*c* space group of the monoclinic crystal system, and it is a high-nuclear spherical cluster. Ho_32_ is composed of a +4 valent cation cluster and four free Cl^−^ ions on the periphery, and its molecular formula is [Ho_32_(L)_24_(*μ*_3_-OH)_48_(*μ*_4_-O)_6_Cl_8_](Cl)_4_·45H_2_O·5CH_3_OH·2CH_3_CN (Table S1). The cationic cluster contains 32 Ho(III) ions, 24 deprotonated ligands L^−^, 48 *μ*_3_-OH^−^ ions formed by the removal of a proton from a water molecule, six *μ*_4_-O^2^^−^ ions formed by the removal of two protons from water molecules, and eight Cl^−^ ions coordinated with end groups (Fig. 1a). It is worth noting that the metal center Ho(III) ions of Ho_32_ are all on the spherical surface, while the ligand L^−^ and the coordinated Cl^−^ ions are both on the outside of the spherical surface. The trigonal {Ln_3_(*μ*_3_-OH)} and square {Ln_4_(*μ*_4_-O)} with shared vertices together form the cluster core of Ho_32_ (Figs 1b, S2a and b). More notably, the peripheral ligand L^−^ tightly wraps the cluster core, further ensuring the stability of Ho_32_ (Fig. 1c and d). Ligand L^−^ is similar to amphiphilic surfactants. The hydrophilic terminal hydroxyl groups coordinate with the metal ions to form a cluster core, while the hydrophobic terminal benzimidazoles are located at the outermost periphery of the cluster, which ensures that the cluster has high stability and good solubility in water. Four eight-coordinated Ho1 and one seven-coordinated Ho2 together constitute the independent unit of Ho_32_, and the eight abovementioned independent units with shared vertices constitute Ho_32_ (Fig. 1e). In addition, we only changed the metal salt to GdCl_3_·6H_2_O and obtained the Gd_32_ homolog of Ho_32_ under the same conditions.

### Stability of Ln_32_ (Ln = Ho and Gd)

**Figure 2.**
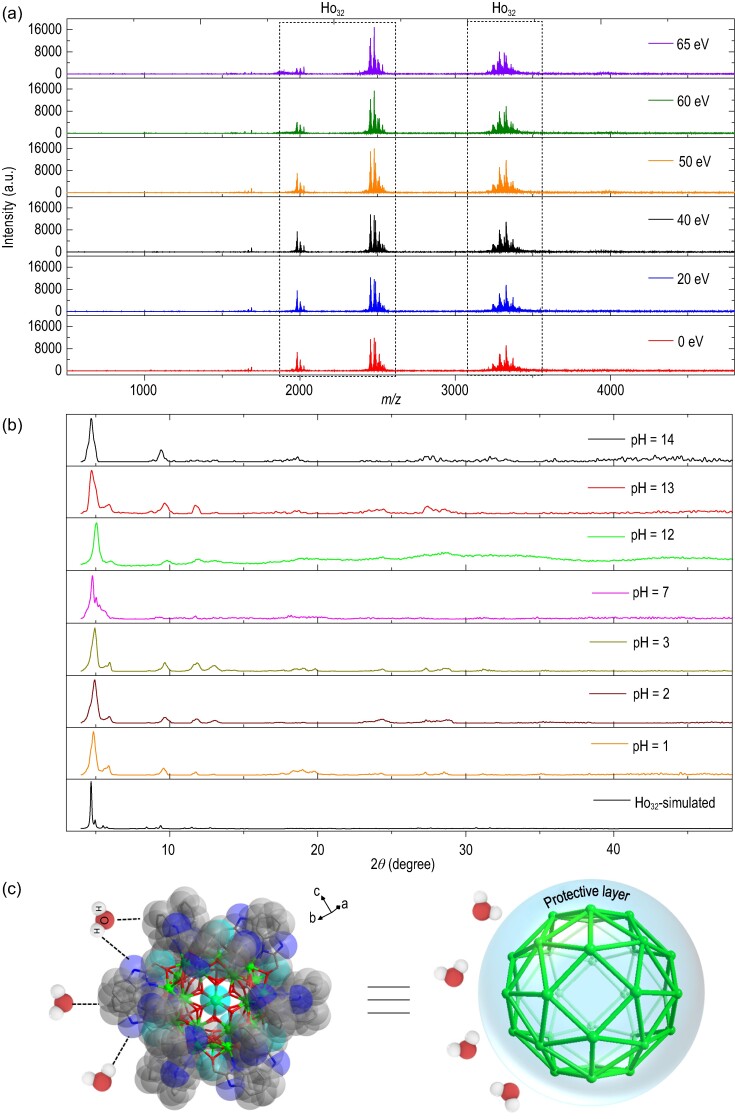
(a) HRESI-MS spectra of Ho_32_ in DMF at different ion source voltages (in-source CID). (b) Comparison of the PXRD observation value and its simulated value after Ho_32_ was immersed in solutions of different pH values for 24 h. (c) The ligands formed a protective layer through hydrogen bonding to prevent H_2_O from attacking the cluster core (the dotted line represents the hydrogen bond).

To explore the functions of a compound, the structure of that compound must be stable [33]. In recent years, HRESI-MS has been widely used to characterize the composition and changes of species in solution, and it has been used to detect the structural stability, fragmentation mechanism, and degree of protonation of clusters [25,33]. The fragment peaks of the Ho_32_ single crystal mass spectrum mainly appear in the range of *m/z* = 1500–3500, and the valence states shown are +3, +4 and +5 (Figs 2a, S8 and S9 and Table S6). Notably, the above molecular ion peaks with different valences are all generated by the main frame Ho_32_, which can be attributed to [Ho_32_L_x_(O)_y_(OH)_z_(solv.)]^3+^ (x = 20 or 21; y = 6; z = 48; solv. = CH_3_OH, CH_3_CN and H_2_O); [Ho_32_L_x_(O)_y_(OH)_z_(solv.)]^4+^ (x = 21 or 22; y = 6; z = 48; solv. = CH_3_OH, CH_3_CN and H_2_O) and [Ho_32_L_x_(O)_y_(OH)_z_(solv.)]^5+^ (x = 22; y = 6; z = 48; solv. = CH_3_CN, H_2_O). As the ion-source voltage gradually increased from 0 eV to 65 eV, Ho_32_ exhibited only molecular ion peaks ([Ho_32_(L)_22_(OH)_48_(O)_6_(Cl)_11_(H^+^)_2_(H_2_O)]^5+^, *m/z* = 1983.35 and [Ho_32_(L)_21_(OH)_48_(O)_6_(Cl)_13_(H^+^)_2_(CH_3_CN)_3_(CH_3_OH)(H_2_O)]^4+^, *m/z* = 2478.69) that coincided with its framework, indicating that it maintained high stability (Fig. S10 and Table S7). Overall, the HRESI-MS test with the Ho_32_ crystal under different energies showed that the crystal has very high stability in solution. Likewise, HRESI-MS indicated that Gd_32_ has high stability in solution (Figs S11 and S12 and Table S8). To verify the water stability of the giant spherical clusters of Ho_32_, they were immersed in aqueous solutions of different pH values (1–14) for 24 h and underwent PXRD testing. It is worth noting that Ho_32_ remains stable in aqueous solutions with different pH values (Fig. 2b). In the Ho_32_ structure, 24 ligands L^−^ wrapped the cluster core and formed a dense protective layer. In addition, 48 *μ*_3_-OH^−^ and 6 *μ*_4_-O^2^^−^ are tightly connected to the metal center through bridging, leading to a highly stable cluster core. When Ho_32_ is attacked by solvent molecules such as H_2_O, the amphiphilic ligand L^−^ (which is similar to surfactants) can effectively resist the attack of solvent molecules through weak supramolecular effects such as hydrogen bonds (Fig. 2c and S13).

### Assembly mechanism of Ho_32_

**Figure 3.**
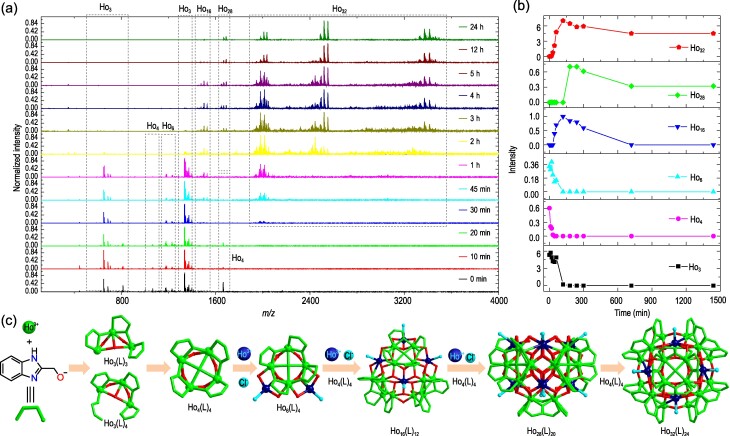
(a) Time-dependent HRESI-MS tracking the formation of Ho_32_. (b) HRESI-MS spectra intensity-time profiles of the species. (c) The possible Ho_32_ assembly mechanism.

HRESI-MS was used to quickly detect the types of molecular ion peaks and their abundance changes in the reaction solution at different time periods to speculate on the most likely self-assembly mechanism of the high-nuclear spherical cluster Ho_32_ (Figs 3a, b and S14, Table S9). Figure 3b shows the time-dependent change trend of the species in the solution during the self-assembly process of Ho_32_. The initial step of the reaction is the combination of Ho(III) ions with the deprotonated ligand (L)^−^, and Ho_3_(L)_3/_Ho_3_(L)_4_ (Ho_3_) are formed by the bridge of *μ*_3_-OH^−^ and *μ*_4_-O^2^^−^ formed with H_2_O. As the self-assembly progresses, Ho_3_(L)_3_/Ho_3_(L)_4_ continuously and rapidly combines with Ho(III) ions and Cl^−^ ions to form intermediates Ho_4_(L)_4_/Ho_4_(L)_5_ (Ho_4_) and Ho_6_(L)_6_/Ho_6_(L)_7_ (Ho_6_), respectively. Then, Ho_6_(L)_4_ is connected with two Ho_4_(L)_4_ molecules through a *μ*_3_-OH^−^ bridge, and the periphery is again combined with Ho(III) ions and Cl^−^ ions to form the intermediate Ho_16_(L)_12_ (Ho_16_). Ho_16_(L)_12_ is connected to two molecules of Ho_4_(L)_4_ through a *μ*_3_-OH^−^ bridge and further combines four molecules of Ho(III) ions and Cl^−^ ions to form Ho_28_(L)_20_. Finally, the apex of Ho_28_(L)_20_ is connected to Ho_4_(L)_4_ through a *μ*_3_-OH^−^ bridge to form a highly symmetric cluster Ho_32_(L)_24_ (Ho_32_). Notably, Ho_32_ is formed by stepwise assembly and template assembly, and Ho_4_(L)_4_ is the template for the self-assembly process. Overall, the possible self-assembly mechanism of Ho_32_ is Ho(III), (L)^−^ and H_2_O → Ho_3_(L)_3_/Ho_3_(L)_4_ → Ho_4_(L)_4_/Ho_4_(L)_5_ → Ho_6_(L)_6_/Ho_6_(L)_7_ → Ho_16_(L)_19_ → Ho_28_(L)_15_ → Ho_32_(L)_24_/Ho_32_(L)_21_/Ho_32_(L)_23_ (Fig. 3c).

### *In vitro* relaxivity and MRI performance of Gd_32_

**Figure 4.**
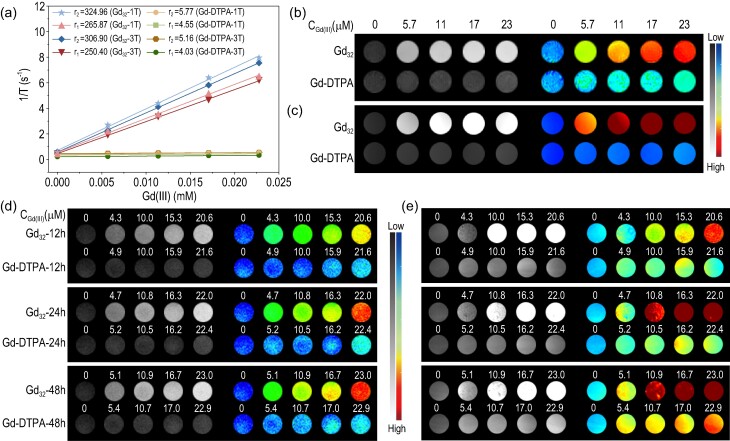
(a) The *r*_1_ and *r*_2_ relaxivities of Gd_32_ and Gd-DTPA solutions containing the same Gd(III) ion concentrations at 1 T and 3 T magnetic fields. Corresponding *T*_1_-weighted MR imaging of Gd(III) ions (Gd_32_ and Gd-DTPA) at 1 T (b) and 3 T (c). *T*_1_-weighted MR imaging of Gd(III) ions (Gd_32_ and Gd-DTPA) incubated with 4T1 cells for different times at 1 T (d) and 3 T (e).

As one of the most established medical imaging techniques, MRI has received great attention [1–7]. The design and synthesis of CAs is essential for obtaining high-resolution and high imaging contrasts. At present, the most commonly used *T*_1_-weighted MRI CAs in clinical practice are gadolinium-based organic chelates, which usually require higher doses to obtain excellent imaging contrasts [13,14]. High-nuclear gadolinium clusters can be highly enriched in Gd(III) in the molecule, so they have great potential for *T*_1_-weighted MRI CAs. To explore the feasibility of Gd_32_ as a *T*_1_-weighted MRI CA, the relaxation time with Gd(III) concentration changes was tested at magnetic field strengths of 1 T and 3 T, and the longitudinal (*r*_1_) and transverse (*r*_2_) relaxation efficiencies were obtained. The results show that the *r*_1_ and *r*_2_ values of Gd_32_ are 265.87 and 324.96 mM^−1^·s^−1^ at a 1 T magnetic field strength and 250.40 and 306.90 mM^−1^·s^−1^ at a 3 T magnetic field strength, respectively (Fig. 4a). Cluster Gd_32_ with highly aggregated Gd(III) shows a higher relaxation value than traditional Gd chelates; *r*_2_/*r*_1_ = 1.22 (*r*_2_/*r*_1_ < 2) indicates that Gd_32_ is a potential candidate for *T*_1_-weighted MRI CAs [16]. As the Gd(III) concentration of Gd_32_ in the aqueous solution gradually increases, the *T*_1_-weighted image gradually becomes brighter with a 1 T magnetic field, and the brightening effect is more obvious with a 3 T magnetic field. In addition, the *T*_1_-weighted pseudo color images displayed by different concentrations of Gd_32_ at 1 T and 3 T both indicate that it has encouraging potential as an MRI CA for biomedical diagnosis (Fig. 4b and c). Cluster Gd_32_ has a good *T*_1_ imaging effect not only in solution but also in cells. Figure 4d shows that as the concentration increased from 0 μM to 23 μM, the *T*_1_-weighted MRI contrast of Gd_32_ and Gd-DTPA on 4T1 cells both increased. However, when the coincubation time was 12 h, 24 h and 48 h, Gd_32_ showed a better *T*_1_-weighted MR imaging effect than Gd-DTPA. Similar results have also been confirmed at a 3 T magnetic field (Fig. 4e). The above data all illustrate the excellent *T*_1_ imaging ability of the high-nuclear gadolinium cluster (Gd_32_) because the molecule can become highly enriched in Gd(III). The large cavity and strong hydrogen bonding with H_2_O lead to the ultrahigh *T*_1_ relaxivity of Gd_32_ (Fig. S15). The parameters obtained from the NMRD fitting results support the high relaxivity of Gd_32_ (Fig. S16 and Table S10). UV−Vis absorption spectroscopy demonstrated that Gd_32_ maintained high stability in PBS, serum (FBS), cell culture medium (DMEM) and PBS solution containing endogenous metal ions (Ca^2+^, Mg^2+^, Fe^3+^, Zn^2+^, *etc.*) (Figs S17 and S18). In addition, Gd_32_ exhibits very low cytotoxicity compared with that of cisplatin, and Gd_32_ can be cleared by the kidney and liver in a short time in mice, which indicates its potential for application in the field of biomedical imaging (Figs S19–S22).

### *In vivo* tumor MRI performance and biodistribution of Gd_32_

**Figure 5.**
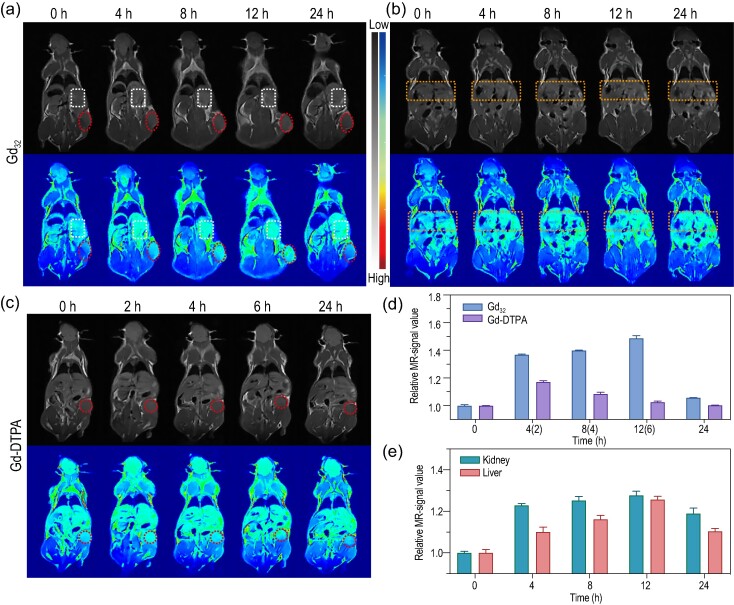
MR imaging *in vivo* at 1 T: after injecting Gd_32_ (a) and the commercial CA Gd-DTPA (c) into BALB/c mice that received 4T1 tumor cells through the tail vein to establish a tumor model, MRI images of the mice at different time points were obtained. The circular frame indicates the tumor site, and the rectangular frame indicates the kidney site. (b) The distribution of Gd_32_ in the liver (oval frame). (d) The relative MR signal values of tumors at different time points in mice injected with Gd_32_ and Gd-DTPA. (e) The relative MR-signal value of the kidney and liver at different time points in a mouse injected with Gd_32_.

To evaluate the effects of Gd_32_ on the MRI of CAs in animal models, we constructed BALB/c mice carrying 4T1 tumors for *in vivo* MRI experiments (Fig. 5). The cluster Gd_32_ was injected into the experimental mice through the tail vein (100 μL, 0.5 mM Gd(III) ions). As shown in Fig. 5a, from 0 h to 12 h, continuous enhancement of *T*_1_ imaging contrast at the tumor site was observed, indicating that Gd_32_ can be effectively enriched at the tumor site. It can be seen from the relative MR signal value of the tumor that at 4 h, Gd_32_ had high enrichment at the tumor site, exhibiting a good *T*_1_ imaging effect. Unlike other small-particle contrast agents that are easily metabolized, Gd_32_ can still achieve good contrast effects 8 h after injection, and the best contrast effects are achieved at 12 h. The signal of the tumor site at 12 h reached 1.49 times the tumor signal of the blank (Fig. 5d). More notably, after injecting Gd_32_ into BALB/c mice bearing 4T1 tumors, the boundaries of tumors can be well distinguished, which is helpful for the subsequent diagnosis of tumors in mice. In addition, as time further increased to 24 h, the *T*_1_ imaging signal of Gd_32_ gradually weakened, indicating that it can be rapidly and effectively metabolized. To compare the MRI effects of Gd_32_ and the clinically used CA Gd-DTPA, the same dose of Gd-DTPA was injected intravenously into BALB/c mice bearing 4T1 tumors under the same conditions. As the time increased from 0 h to 2 h, the *T*_1_ imaging effect of Gd-DTPA at the tumor site gradually increased, and the best *T*_1_ imaging effect was achieved at 2 h (Fig. 5c); however, at 2 h, the signal at the tumor site was only 1.17 times the tumor signal in the blank (Fig. 5d). The above data show that under the condition of the same extremely small dose of CAs, Gd_32_ shows a far better *T*_1_-weighted MRI imaging effect than Gd-DTPA. Similar results appeared in 3 T MRI (Fig. S23). Excellent MRI CAs can be effectively and rapidly metabolized, which prevents the toxicity and enrichment of heavy metal ions in living bodies. Therefore, we explored the metabolism and biodistribution of Gd_32_ in mice. After injecting Gd_32_ through the tail vein, *T*_1_-weighted MRI images of the mouse kidney and liver were collected at different time points (0, 4, 8, 12 and 24 h) (Fig. 5a and b). As shown in Fig. 5a, as the injection time increased from 0 h to 12 h, the enrichment of Gd_32_ in the kidney gradually increased, and the yellow−green color gradually increased also. It is worth noting that the *T*_1_-weighted MRI image of Gd_32_ on the kidney gradually weakened after the injection time was further increased to 24 h, indicating that it was gradually metabolized. In addition, monitoring the intensity changes in MRI images of the liver of mice after injection of Gd_32_ at different time points obtained similar results, which shows that the liver can also metabolize part of Gd_32_. In general, the content of Gd_32_ in the kidneys of mice is higher than that in the liver, indicating that ultrasmall Gd_32_ is mainly eliminated by the kidney (Fig. 5e) [34]. Although Gd_32_ with high positive charge that is diluted by mouse body fluids and easily combined with negatively charged biological macromolecules in the body, resulting in *in vivo* imaging effects that are not as good as those of solutions, there are still obvious *T*_1_ imaging effects *in vivo*. The above results indicate that Gd_32_ is an ideal candidate for MRI CAs and has great application prospects in clinical tumor diagnosis. In addition, high-nuclear Gd clusters with highly aggregated Gd(III) at the molecular level have higher imaging contrast than traditional Gd chelates, which effectively prevents the need to use large doses of traditional CAs.

## CONCLUSIONS

In summary, we report two spherical high-nucleus lanthanide nanoclusters with high stability in solution. Crystallography, HRESI-MS and PXRD jointly confirmed the high stability of Ho_32_ under solution conditions. Time-dependent HRESI-MS tracked the formation process of Ho_32_, a variety of different types of intermediates were screened, and the gradual assembly formation mechanism of spherical clusters was proposed for the first time. The excellent water solubility and water stability of Gd_32_ prompted us to explore its potential applications in the field of biomedicine. Notably, Gd_32_, a nanocluster with low toxicity, high biocompatibility, and a high relaxation rate, shows excellent *T*_1_-weighted MRI effects at the cell and animal levels. The gadolinium-based nanocluster Gd_32_ with highly aggregated Gd(III) has significantly better MRI imaging contrast than the clinically used commercial CA Gd-DTPA, which effectively prevents the need to use a large dose of traditional gadolinium contrast agents. To the best of our knowledge, this is the first study to explore the application of high-nuclear lanthanide clusters in the field of MRI. This work provides a detailed example of the construction of lanthanide clusters with high stability and high water solubility. In addition, this work also opens a door to study the performance of lanthanide clusters in solution.

## MATERIALS AND METHODS

### Synthesis of Ln_32_

A mixture of HL (0.1 mmol, 148 mg), HoCl_3_⋅6H_2_O (0.5 mmol, 189.7 mg) and 250 μL TEA was dissolved in 5 mL MeOH and 5 mL MeCN. Then, the solution was stirred for 0.5 h. Next, the solution was transferred to a Teflon container in a stainless-steel bomb and kept at 100°C in the oven for 48 h. The solution was then filtered and allowed to stand until evaporation. Yellow crystals of Ho_32_ were collected (yield, 35 mg, 23.6% based on ligand HL). The synthetic method of Gd_32_ is the same as that of Ho_32_, only changing HoCl_3_⋅6H_2_O to GdCl_3_⋅6H_2_O. Yellow crystals were collected (yield, 25 mg, 16.9% based on ligand HL).

## Supplementary Material

nwad036_Supplemental_File
