# Supplementary material for: High-stability spherical lanthanide nanoclusters for magnetic resonance imaging
Source: Natl Sci Rev. 2023 Feb 16;10(4):nwad036. doi: 10.1093/nsr/nwad036 (PMC10187785; doi:10.1093/nsr/nwad036)
Supplement: nwad036_Supplemental_File [file nwad036_supplemental_file.docx]

Supporting Information

**High-Stability** **Spherical Lanthanide Nanoclusters for Magnetic Resonance Imaging**

Hai-Ling Wang^⊥,†^, Dong-Lin Liu^⊥,‡^, Jian-Hua Jia^†^, Jun-Liang Liu^†^, Ze-Yu Ruan^†^, Wei Deng^†^, Shi-Ping Yang^*,‡^, Si-Guo Wu^†^, Ming-Liang Tong^*,†^

^†^Key Laboratory of Bioinorganic and Synthetic Chemistry of Ministry of Education, School of Chemistry, Sun Yat-Sen University, 510275 Guangzhou, Guangdong, P. R. China

^‡^College of Chemistry and Materials Science, Shanghai Normal University, Shanghai, China

^⊥^These authors contributed equally to this work.

*E-mail: tongml@mail.sysu.edu.cn (M. L. Tong); shipingy@shnu.edu.cn (S. P. Yang)

**Contents:**

[**Experimental Section** 5](#_Toc116903073)

[**Table S1.** Crystallographic data of the clusters **Ho_32_** and **Gd_32_**. 9](#_Toc116903074)

[**Table S2.** Selected bond lengths (Å) and angles (°) of **Ho_32_**. 10](#_Toc116903075)

[**Table S3.** Selected bond lengths (Å) and angles (°) of **Gd_32_**. 11](#_Toc116903076)

[**Table S4.** *SHAPE* analysis of the Ho1 for cluster **Ho_32_**. 12](#_Toc116903077)

[**Table S5.** *SHAPE* analysis of the Ho2 for cluster **Ho_32_**. 12](#_Toc116903078)

[**Figure S1.** The crystal photo of the spherical cluster **Ho_32_**. 13](#_Toc116903079)

[**Figure S2.** Structure of trigonal {Ln_3_(*μ*_3_-OH)} (a) and square {Ln_4_(*μ*_4_-O)} (b); (c) Coordination mode of ligand L^-^. 13](#_Toc116903080)

[**Figure S3.** Metal coordination configuration of Ho1 (a) and Ho2 (b). 14](#_Toc116903081)

[**Figure S4.** Molecular structure (a), metal connection (b) and space-filling mode (c) of the cluster [Gd_32_(OH)_54_(mda)_12_(NO_3_)_12_(H_2_O)_24_⊃(H_2_O)_6_](OH)_6_ (for clarity, H atom, free OH^-^ ions and solvent molecules have been omitted).^5^ 15](#_Toc116903082)

[**Figure S5.** Infrared spectra (IR) of clusters **Ho_32_** (a) and **Gd_32_** (b). 16](#_Toc116903083)

[**Figure S6.** TG curve of clusters **Ho_32_** (a) and **Gd_32_** (b). 16](#_Toc116903084)

[**Figure S7.** Powder diffraction pattern (PXRD) of clusters **Ho_32_** and **Gd_32_**. 17](#_Toc116903085)

[**Figure S8.** Positive HRESI-MS spectra of **Ho_32_** in DMF. 17](#_Toc116903086)

[**Table S6.** Major species assigned in the HRESI-MS of **Ho_32_** in positive mode. 18](#_Toc116903087)

[**Figure S9.** The superposed simulated and observed spectra of several species for cluster **Ho_32_.** 19](#_Toc116903088)

[**Table S7.** Major species assigned in the HRESI-MS of **Ho_32_** with different in-source CID (0-65 eV) in positive mode. 19](#_Toc116903089)

[**Figure S10.** The superposed simulated and observed spectra of several species for **Ho_32_** with different in-source CID (0-65 eV). 20](#_Toc116903090)

[**Table S8.** Major species assigned in the HRESI-MS of **Gd_32_** in positive mode. 20](#_Toc116903091)

[**Figure S11.** Positive HRESI-MS spectra of **Gd_32_** in DMF. 21](#_Toc116903092)

[**Figure S12.** The superposed simulated and observed spectra of several species for cluster **Gd_32_**. 22](#_Toc116903093)

[**Figure S13.** (a) Schematic diagram of surfactant-coated high-nuclear clusters;^6,7^ (b) The flow chart of using amphiphilic ligands to synthesize high-nuclear rare earth clusters with high water stability and water solubility. 23](#_Toc116903094)

[**Table S9.** Major species assigned in the time-dependent HRESI-MS for tracked the formation of **Ho_32_** in positive mode. 24](#_Toc116903095)

[**Figure S14.** The time-dependent HRESI-MS superposed simulated and observed spectra of several species for tracked the formation of **Ho_32_**. 27](#_Toc116903096)

[**Figure S15.** Pictorial description of the parameters that influence the relaxivity of **Gd_32_** (H atom, free Cl^-^ ions and solvent molecules have been omitted for clarity). 29](#_Toc116903097)

[**Figure S16.** Longitudinal relaxivity distributions of cluster Gd_32_ under different field strengths (different hydrogen proton Larmor frequencies), and fitting curves based on Solomon-Bloembergen Morgan (SBM) paramagnetic relaxation theory. 30](#_Toc116903098)

[**Table S10.** Parameters during longitudinal relaxation of **Gd-DTPA** and **Gd_32_**. 30](#_Toc116903099)

[**Figure S17.** UV-Vis absorption spectra of **Gd_32_** in PBS, serum (FBS), cell culture medium (DMEM and DMEM^++^, DMEM^++^ denotes DMEM medium containing penicillin and streptomycin) and PBS solution containing endogenous metal ions (Ca^2+^, Mg^2+^, Fe^3+^, Zn^2+^, *etc.*). 30](#_Toc116903100)

[**Figure S18.** UV-Vis absorption spectra of **Gd_32_** in aqueous solutions of different pH (1-14). 31](#_Toc116903101)

[**Figure S19.** Cell viability for HUVEC and 4T1 cells after incubation with cluster **Gd_32_** (a, c) and cisplatin (b, d) for 12 h and 24 h, respectively. 31](#_Toc116903102)

[**Figure S20.** (a) Cell viability of HUVECs after incubation with **Gd_32_** and cisplatin for 12 h and 24 h. (b) Red blood cells incubated with various concentrations of **Gd_32_** for 12 h (PBS and pure water were employed as the negative and positive controls, respectively). (c) Body weight changes of mice in the intravenously injected **Gd_32_** at a dose of 10 mg/kg and mice in the noninjected group (blank) within one week. (d) Routine blood tests of healthy mice and mice injected with **Gd_32_**. (e) Histopathological examination of the main organs of mice injected with PBS (control) and **Gd_32_**, respectively (Scale bar: 100 µm). 32](#_Toc116903103)

[**Figure S21.** Comparison of the cytotoxicity of cluster **Gd_32_** and cisplatin after co-incubation with 4T1 cells for 12 h and 24 h, respectively. 33](#_Toc116903104)

[**Figure S22.** Ultraviolet-visible (UV-Vis) absorption spectrum of hemolysis experiments with cluster **Gd_32_** of different concentrations. 33](#_Toc116903105)

[**Figure S23.** MR-imaging in vivo at 3 T: After injecting cluster **Gd_32_** (a) and commercial contrast agents Gd-DTPA (b) into BALB/c mice carrying the 4T1 tumor model through the tail vein, the MRI images of the mice at different time points, the circular frame is the tumor site; (c) The relative MR-signal value of tumors at different time points of the mice injected with cluster **Gd_32_** and commercial contrast agents Gd-DTPA, respectively. 34](#_Toc116903106)

[**Reference** 35](#_Toc116903107)

# Experimental Section

**Materials and general procedures.**

Metal salts and other reagents were commercially available and used as received without further purification. The C, H, N elemental analyses were carried out with an Elementar Vario-EL CHNS elemental analyzer. Powder X-ray diffraction (PXRD) patterns were performed on Bruker D8 Advance Diffratometer (Cu-Kα, λ = 1.54056 Å). Thermogravimetric analysis (TGA) was carried out on a NETZSCH TG209F3 thermogravimetric analyzer. The ICP-AES analyses were carried on a TJA IRIS (HR) spectrometer. The IR (KBr pellet) spectrum was recorded (500-4000 cm^-1^ region) on a NICOLET iS10 FT-IR spectrometer. Magneto relaxivity were measured with 1 T magnetic resonance instrument (NMI20-Analyst, Shanghai Niumag Corporation) and 3 T magnetic resonance instrument (Magnetom Prisma, Siemens). For in vitro imaging, the parameters were TE = 0.04 ms and TR = 200 ms. And for in vivo MR imaging were estimated using the following parameters: slice thickness of 3 mm; a TE of 18.125 ms; a TR of 340 ms; field of view, 80 × 80 mm; matrix size, 256 × 192 mm. MTT absorption intensity were measured by used a microplate reader (RNE-90002, Reagen, USA). 1H NMRD was performed on 0.25 Tesla Bench-top Fast Field Cycling NMR Relaxometer (SMARtracer™).

**Single crystal X-ray crystallography.**

Diffraction data were collected on a Bruker D8 QUEST diffractometer with Mo-*K*_α_ radiation (λ = 0.71073 Å) for all complexes at 120 K. The Data indexing and integration were carried out using a Bruker Smart program. The structures were solved by direct methods, and all non-hydrogen atoms were refined anisotropically by least-squares on *F*^2^ using the SHELXTL program suite and OLEX2.^1^ Anisotropic thermal parameters were assigned to all non-hydrogen atoms. The hydrogen atoms attached to carbon, nitrogen and oxygen atoms were placed in idealized positions and refined using a riding model to the atom to which they were attached. The SQUEEZE program of PLATON was employed to deal with the disordered solvent molecules.^2^ The CCDC reference numbers are 2103382 (**Ho_32_**) and 2103377 (**Gd_32_**). These data can be obtained free of charge via <https://www.ccdc.cam.ac.uk/structures/>.

**HRESI-MS measurement.**

HRESI-MS measurements were conducted at a capillary temperature of 275 °C. Aliquots of the solution were injected into the device at 2 μL. The mass spectrometer used for the measurements was a Bruker, and the data were collected in positive and negative ion modes. The spectrometer was previously calibrated with the standard tune mix to give a precision of *ca*. 2 ppm within the region of 1500−3,500 *m/z*. The capillary voltage was 50 V, the tube lens voltage was 150 V, and the skimmer voltage was 25 V. The in-source energy was set within the range of 0-65 eV with a gas flow rate at 10% of the maximum.

**Tracking of the assembly process.**

A mixture of HL (0.1 mmol, 148 mg), HoCl_3_⋅6H_2_O (0.5 mmol, 189.7 mg) and 250 μL TEA were dissolved in 5 mL MeOH and 5 mL MeCN. For the above experiments, multiple groups of parallel control groups under the same conditions were carried out, and the above control groups were all heated in a 100 °C oven. At the set reaction time points, specific control groups were taken out and rapidly cooled down for HRESI-MS sample preparation and detection.

**ICP testing of Gd_32_ and Gd-DTPA.**

**Gd_32_**: Accurately weigh 27.32 mg of the **Gd_32_** crystals sample and dissolve it in 250 μL of DMSO, and add 2750 μL of ultrapure water to make a solution with a total volume of 3 mL. Take 10 μL of the above 3 mL solution, and add 500 μL of nitric acid to completely digest it, and finally add 9490 μL of ultrapure water to make up to 10 mL for ICP testing. The mass concentration of **Gd_32_** in the solution before the final test was 9.11 mg/L. Several groups of parallel experiments were carried out, and the results are as follows:

| Numbers | Gd(III) (mg/L) |
| --- | --- |
| **Gd_32_**-1 | 4.01 |
| **Gd_32_**-2 | 3.89 |
| **Gd_32_**-3 | 4.00 |
| **Gd_32_**-4 | 4.00 |
| Average value | 3.975 |

Convert mass concentration to molar concentration: **Gd_32_** : Gd(III) ions = 9.11*10^-3^ (g/L) / 10806.43 (g/moL) : 3.975*10^-3^ (g/L) / 157.25 (g/moL) = 1 : 30.

**Gd-DTPA**: 5.21 mg of Gd-DTPA (CAS: 86050-77-3) solid sample was accurately weighed and dissolved in 250 μL of DMSO, and 750 μL of ultrapure water was added to make a total volume of 1 mL. Take 10 μL from the above 1 mL solution, and add 500 μL of nitric acid to completely digest it, and finally add 9490 μL of ultrapure water to make up to 10 mL for ICP test. The mass concentration of Gd-DTPA in the solution before the final test was 5.21 mg/L. Several groups of parallel experiments were carried out, and the results are as follows:

| Numbers | Gd(III) (mg/L) |
| --- | --- |
| **Gd-DTPA-1** | 0.876 |
| **Gd-DTPA-2** | 0.897 |
| **Gd-DTPA-3** | 0.862 |
| **Gd-DTPA-4** | 0.858 |
| Average value | 0.8733 |

Convert mass concentration to molar concentration: Gd-DTPA : Gd(III) ions = 5.21*10^-3^ (g/L) / 938 (g/moL) : 0.8733*10^-3^ (g/L) / 157.25 (g/moL) = 1 : 1.

**Solution MRI imaging experiments.**

**Gd_32_**: Prepare 1 mL of **Gd_32_** solution with a concentration of 0.19 mM (the content of DMSO is less than 1%, which is used for solubilization). After conversion of ICP results (**Gd_32_** : Gd(III) ions = 1 : 30), the molar concentration of Gd(III) ions in the above-mentioned **Gd_32_** mother liquor was obtained as 5.7 mM. For solution MRI imaging experiments, the above-mentioned solutions containing Gd(III) ions at a concentration of 5.7 mM were diluted to 0.0057, 0.011, 0.017, and 0.023 mM (5.7, 11, 17, and 23 μM), respectively.

**Gd-DTPA**: Prepare 1 mL of Gd-DTPA solution with a concentration of 5.7 mM (the content of DMSO is less than 1%, which is used for solubilization). After conversion of ICP results (Gd-DTPA : Gd(III) ions = 1 : 1), the molar concentration of Gd(III) ions in the above-mentioned Gd-DTPA mother liquor was obtained as 5.7 mM. For solution MRI imaging experiments, the above-mentioned solutions containing Gd(III) ions at a concentration of 5.7 mM were diluted to 0.0057, 0.011, 0.017, and 0.023 mM (5.7, 11, 17, and 23 μM), respectively.

The above experimental groups of **Gd_32_** and Gd-DTPA with different concentrations were used for relaxation rate tests and their *T*_1_-weighted images were collected.

**Phagocytosis assay and cell MRI imaging.**

In cells, Gd(III) ions concentration of 0, 0.057, 0.011, 0.017 and 0.023 mM was incubated with 4T1 cells for 12, 24 and 48 h, respectively. Then, the cell supernatant was removed and adherent cells were collected and dispersed in 1 mg/mL xanthan gum with the same volume of medium used to incubate the cells. Gd(III) ions phagocytosed cells suspended in xanthan gum were used for the test or MRI performance and the concentration of phagocytosed Gd(III) ions was measured by ICP-MS. Then the *T*_1_-weighed images at different times was collected by the parameter in materials and general procedures.

**ICP-MS testing of the concentration of phagocytosed Gd(III) ions in cells.**

Take 2 mL of the above xanthan gum solution dispersed with phagocytic Gd(III) cells, add 2 mL of nitric acid, and let stand for 48 hours until the digestion is complete, and then for ICP-MS testing. The results are as follows (Since the concentration is diluted during the digestion, the actual concentration in the following table is the detection concentration multiplied by 2)：

| Numbers | C (mg/L) | C (μM) | Numbers | C (mg/L) | C (μM) |
| --- | --- | --- | --- | --- | --- |
| **Gd_32_-5.7-12 h** | 0.6820 | 4.3 | **Gd-DTPA-5.7-12 h** | 0.7693 | 4.9 |
| **Gd_32_-5.7-24 h** | 0.7424 | 4.7 | **Gd-DTPA-5.7-24 h** | 0.8207 | 5.2 |
| **Gd_32_-5.7-48 h** | 0.7979 | 5.1 | **Gd-DTPA-5.7-48 h** | 0.8507 | 5.4 |
| **Gd_32_-11-12 h** | 1.5768 | 10.0 | **Gd-DTPA-11-12 h** | 1.5725 | 10.0 |
| **Gd_32_-11-24 h** | 1.7053 | 10.8 | **Gd-DTPA-11-24 h** | 1.6589 | 10.5 |
| **Gd_32_-11-48 h** | 1.7111 | 10.9 | **Gd-DTPA-11-48 h** | 1.6898 | 10.7 |
| **Gd_32_-17-12 h** | 2.4038 | 15.3 | **Gd-DTPA-17-12 h** | 2.5004 | 15.9 |
| **Gd_32_-17-24 h** | 2.5684 | 16.3 | **Gd-DTPA-17-24 h** | 2.5445 | 16.2 |
| **Gd_32_-17-48 h** | 2.6253 | 16.7 | **Gd-DTPA-17-48 h** | 2.6711 | 17.0 |
| **Gd_32_-23-12 h** | 3.2377 | 20.6 | **Gd-DTPA-23-21 h** | 3.3978 | 21.6 |
| **Gd_32_-23-24 h** | 3.4651 | 22.0 | **Gd-DTPA-23-24 h** | 3.5169 | 22.4 |
| **Gd_32_-23-48 h** | 3.6168 | 23.0 | **Gd-DTPA-23-48 h** | 3.6045 | 22.9 |

**In vivo MRI imaging.**

A PBS solution of **Gd_32_** having a concentration of 0.5 mM was prepared and diluted 30-fold with PBS to obtain a solution having a Gd(III) ion concentration of 0.5 mM. A PBS solution containing the commercial MRI contrast agent Gd-DTPA with a Gd(III) ions concentration of 0.5 mM was prepared. For in vivo MRI imaging, a dose of 100 μL of the above solution was injected into mice through the tail vein. Then the *T*_1_-weighed images at different times was collected by the parameter in materials and general procedures.

**The stability of Gd_32_ was tested by UV-Vis absorption spectroscopy.**

Use DMSO to dissolve **Gd_32_** crystals, and were added to H_2_O, PBS, serum (FBS), cell culture medium (DMEM and DMEM^++^, DMEM^++^ denotes DMEM medium containing penicillin and streptomycin) and PBS solution containing endogenous metal ions (Ca^2+^, Mg^2+^, Fe^3+^, Zn^2+^, *etc.*) (the content of DMSO is less than 1%).^3^ The above solution containing **Gd_32_** at a concentration of 5 μM was finally obtained. Since the absorption values of serum and cell culture medium in the ultraviolet region have a certain overlap with **Gd_32_**, the above solutions without **Gd_32_** were used as control groups for baseline correction. Finally, the above solution was tested by UV-Vis absorption spectroscopy (Figure S16).

**Cytotoxicity Assay.**

The cytotoxicity of the coordination complexes toward both human umbilical vein endothelial cells (HUVECs), and mouse breast cancer cells (4T1) was studied by the classic MTT assay. The cells were cultured in an incubator with a temperature of 37 °C and a CO_2_ content of 5% to grow adherently. The cells were incubated together for 12 h and 24 h of cluster **Gd_32_** and cisplatin with different concentrations. The cell viability was calculated on the basis of the MTT absorption intensity, as measured using a microplate reader (Varioskan Flash, Thermo Fisher Scientific). Hemolysis assays were carried out on red blood cells (RBCs, obtained from healthy BALB/c nude mice 5−6 weeks old; Shanghai Laboratory Animal Center). RBCs (0.4 mL, 2% volume ratio) was added to 1 mL deionized water, PBS, or PBS mixtures containing cluster **Gd_32_** with different concentrations, respectively. After 4 hour’s incubations at 37 °C, mixtures were centrifuged at 3,000 rpm for 5 minutes. Further, to evaluate cytotoxicity of cluster **Gd_32_** to mice, histological analysis, blood routine examination and biochemical index were performed. The control group (three healthy mice) and treatment group (injection of cluster **Gd_32_** at a dose of 10 mg/kg for 7 days) were humanely sacrificed and dissected. At the same time, the weight for the two groups of mice was monitored every day.

**Synthetic part.**

**Synthesis of [Ho_32_(L)_24_(*μ*_3_-OH)_48_(*μ*_4_-O)_6_Cl_8_](Cl)_4_∙45H_2_O∙5CH_3_OH∙2CH_3_CN (Ho_32_).**

A mixture of HL (0.1 mmol, 148 mg), HoCl_3_⋅6H_2_O (0.5 mmol, 189.7 mg) and 250 μL TEA were dissolved in 5 mL MeOH and 5 mL MeCN. Then, the solution was stirred for 0.5 h. Then solution was transferred to a Teflon container in stainless steel bomb and kept at 100 °C in the oven for 48 h. After that, the solution was filtered and kept still for evaporation. Yellow crystals were collected (Yield, 35 mg, 23.6% based on ligand HL). Elemental analysis calc. (%) for C_201_H_332_Cl_12_Ho_32_N_50_O_128_; C: 21.55, H: 2.99, N: 6.25; found (%): C: 21.61, H: 2.75, N: 5.96.

**Synthesis of [Gd_32_(L)_24_(*μ*_3_-OH)_48_(*μ*_4_-O)_6_Cl_8_](Cl)_4_∙35H_2_O∙6CH_3_OH∙2CH_3_CN (Gd_32_).**

A mixture of HL (0.1 mmol, 148 mg), GdCl_3_⋅6H_2_O (0.5 mmol, 185.9 mg) and 250 μL TEA were dissolved in 5 mL MeOH and 5 mL MeCN. Then, the solution was stirred for 0.5 h. Then solution was transferred to a Teflon container in stainless steel bomb and kept at 100 °C in the oven for 48 h. After that, the solution was filtered and kept still for evaporation. Yellow crystals were collected (Yield, 25 mg, 16.9% based on ligand HL). Elemental analysis calc. (%) for C_202_H_316_Cl_12_Gd_32_N_50_O_119_; C: 22.45, H: 2.95, N: 6.48; found (%): C: 22.29, H: 2.75, N: 6.29.

# Table S1. Crystallographic data of the clusters Ho_32_ and Gd_32_.

| **Complexes** | **Ho_32_** | **Gd_32_** |
| --- | --- | --- |
| Formula | C_201_H_332_Cl_12_Ho_32_N_50_O_128_ | C_202_H_316_Cl_12_Gd_32_N_50_O_119_ |
| Formula weight | 11200.31 | 10806.43 |
| *T* (K) | 120 K | 119.99 K |
| Crystal system | Monoclinic | Monoclinic |
| Space group | *C*2/*c* | *C*2/*c* |
| *a* (Å) | 30.7424 (11) | 31.1465 (8) |
| *b* (Å) | 35.6312 (13) | 35.8636 (11) |
| *c* (Å) | 32.1380 (12) | 32.5328 (9) |
| *α* (°) | 90° | 90° |
| *β* (°) | 90.446 (2) | 90.1136 (14) |
| *γ* (°) | 90° | 90° |
| *V* (Å^3^) | 35203 (2) | 36339.9(18) |
| *Z* | 4 | 4 |
| *D*_c_ (g cm^–3^) | 2.114 | 1.975 |
| *μ* (mm^–1^) | 7.27 | 5.912 |
| Reflns coll. | 104445 | 40906 |
| Unique reflns | 31952 | 40906 |
| R_int_ | 0.062 | - |
| ^a^*R*_1_ [*I* ≥ 2*σ*(*I*)] | 0.069 | 0.075 |
| ^b^*wR*_2_ (all data) | 0.194 | 0.224 |
| *GOF* | 1.06 | 1.048 |

^a^*R*_1_=Σ||*F*_o_|—|*F*_c_||/Σ|F_o_|, ^b^w*R*_2_=[Σw(*F*_o_^2^—*F*_c_^2^)^2^/Σw(*F*_o_^2^)^2^]^1/2^.

# Table S2. Selected bond lengths (Å) and angles (°) of Ho_32_.

| **Bond lengths (Å)** | | | | | | |
| --- | --- | --- | --- | --- | --- | --- |
| Ho1-O8 | 2.298 (10) | Ho2-O7 | 2.283 (10) | Ho3-O11 | 2.284 (11) | |
| Ho1-O12 | 2.277 (11) | Ho2-O30 | 2.557 (11) | Ho3-O17 | 2.321 (10) | |
| Ho1-O14 | 2.509 (11) | Ho2-O17 | 2.334 (11) | Ho3-O18 | 2.329 (11) | |
| Ho6-O22 | 2.373 (10) | Ho2-N10 | 2.451 (14) | Ho3-O27 | 2.317 (9) | |
| Ho6-O15 | 2.372 (10) | Ho5-N18 | 2.408 (15) | Ho4-O12 | 2.280 (11) | |
| Ho6-N14 | 2.411 (13) | Ho5-O9 | 2.275 (11) | Ho4-O10 | 2.244 (11) | |
| Ho6-O8 | 2.255 (11) | Ho5-O10 | 2.266 (11) | Ho4-O11 | 2.394 (11) | |
| Ho7-O15 | 2.333 (11) | Ho5-O14 | 2.514 (11) | Ho4-O19 | 2.442 (10) | |
| Ho7-O23 | 2.304 (11) | Ho5-O21 | 2.371 (10) | Ho4-N22 | 2.452 (13) | |
| Ho7-O25 | 2.297 (11) | Ho8-O1 | 2.254 (10) | Ho9-O7 | 2.278 (9) | |
| Ho7-O16 | 2.320 (10) | Ho8-O13 | 2.306 (10) | Ho9-O30 | 2.523 (9) | |
| Ho7-O24 | 2.338 (9) | Ho8-O36 | 2.447 (10) | Ho9-O27 | 2.359 (10) | |
| Ho10-O2 | 2.267 (10) | Ho11-O2 | 2.298 (11) | Ho12-O34 | 2.294 (10) | |
| Ho10-O25 | 2.442 (9) | Ho11-O3 | 2.276 (10) | Ho12-O21 | 2.343 (9) | |
| Ho10-O36 | 2.371 (10) | Ho11-O24 | 2.339 (11) | Ho14-O20 | 2.300 (11) | |
| Ho15-O19 | 2.373 (10) | Ho15-O5 | 2.278 (12) | Ho14-O19 | 2.300 (11) | |
| Ho16-O5 | 2.279 (11) | Ho15-O32 | 2.538 (2) | Ho14-O33 | 2.355 (9) | |
| Ho16-O6 | 2.279 (9) | Ho16-O28 | 2.404 (10) | Ho16-O29 | 2.368 (10) | |
| **Bond angles (°)** | | | | | | |
| O18-Ho1-O17 | 68.7 (4) | O26-Ho2-O30 | 72.0 (3) | O28-Ho3-Cl4 | | 82.5 (3) |
| O18-Ho1-O14 | 73.0 (3) | O26-Ho2-O27 | 77.1 (4) | O28-Ho3-O18 | | 145.6 (4) |
| O18-Ho1-N12 | 134.0 (4) | O26-Ho2-N10 | 135.2 (4) | O28-Ho3-O17 | | 119.2 (4) |
| O8-Ho1-O15 | 71.8 (4) | O17-Ho2-O26 | 112.8 (4) | O28-Ho3-O29 | | 73.1 (4) |
| O8-Ho1-O16 | 86.1 (4) | O17-Ho2-O30 | 143.1 (3) | O28-Ho3-O27 | | 72.2 (4) |
| O20-Ho4-N22 | 132.5 (4) | O20-Ho5-O14 | 73.0 (3) | O22-Ho6-O14 | | 142.2 (4) |
| O12-Ho4-O20 | 137.8 (4) | O10-Ho5-O21 | 137.2 (4) | O15-Ho6-O14 | | 72.4 (3) |
| O10-Ho4-O14 | 72.5 (4) | O10-Ho5-N18 | 84.6 (5) | O23-Ho6-O14 | | 139.2 (3) |
| O11-Ho4-O19 | 70.8 (4) | O9-Ho5-O20 | 138.4 (4) | O9-Ho6-O22 | | 83.9 (4) |
| O18-Ho4-O20 | 78.9 (4) | O34-Ho5-O14 | 139.1 (3) | N14-Ho6-O14 | | 119.5 (4) |
| O25-Ho7-O15 | 145.2 (4) | O13-Ho8-O36 | 149.3 (4) | O28-Ho9-N4 | | 80.5 (4) |
| O16-Ho7-Cl1 | 84.4 (3) | N6-Ho8-O30 | 118.2 (4) | O7-Ho9-O30 | | 71.5 (4) |
| O24-Ho7-O26 | 81.2 (4) | O1-Ho8-O30 | 71.0 (4) | O27-Ho9-O30 | | 73.8 (3) |
| O23-Ho7-O16 | 119.1 (4) | O36-Ho8-O30 | 138.5 (3) | O27-Ho9-N4 | | 134.8 (4) |
| O15-Ho7-O24 | 81.3 (4) | O25-Ho8-O36 | 69.9 (3) | O7-Ho9-O27 | | 71.3 (4) |
| O38-Ho10-O35 | 72.8 (3) | O23-Ho11-N16 | 86.2 (4) | O22-Ho12-O21 | | 73.4 (3) |
| O36-Ho10-N8 | 87.1 (5) | O2-Ho11-O22 | 149.3 (4) | O21-Ho12-Cl2 | | 131.7 (3) |
| O24-Ho10-O35 | 72.6 (3) | O3-Ho11-N16 | 70.8 (4) | O34-Ho12-O22 | | 118.1 (4) |
| O25-Ho10-O35 | 138.9 (3) | O24-Ho11-O22 | 113.5 (3) | O34-Ho12-O21 | | 72.9 (4) |
| O2-Ho10-O24 | 71.4 (3) | O24-Ho11-O23 | 71.7 (3) | O34-Ho12- Cl2 | | 83.0 (3) |
| O33-Ho13-O34 | 112.4 (4) | O19-Ho14-O20 | 73.4 (4) | O19-Ho15-O32 | | 140.2 (3) |
| O4-Ho13-O33 | 70.4 (4) | O20-Ho14-O33 | 81.7(4) | O11-Ho15-O32 | | 139.9 (3) |
| O34-Ho13-N20 | 86.4 (4) | O19-Ho14-O33 | 146.5 (4) | O11-Ho15-N24 | | 79.0 (4) |
| O4-Ho13-N20 | 72.0 (4) | O20-Ho14- Cl3 | 131.7 (3) | O19-Ho15-O11 | | 70.7 (4) |
| O4-Ho13-O34 | 146.2 (3) | O33-Ho14-Cl3 | 131.2 (3) | O5-Ho15-O19 | | 148.6 (4) |
| O31-Ho16-O32 | 140.6 (3) | O37-Ho16-O31 | 70.5 (4) | O28-Ho16-N2 | | 87.4 (4) |
| O29-Ho16-O31 | 113.0 (4) | O6-Ho16-O29 | 139.6 (4) | O5-Ho16-O28 | | 82.5 (4) |
| Symmetry code: (i) 1-X,+Y,3/2-Z | | | | | | |

#

# Table S3. Selected bond lengths (Å) and angles (°) of Gd_32_.

| **Bond lengths (Å)** | | | | | | |
| --- | --- | --- | --- | --- | --- | --- |
| Gd1-O13 | 2.361 (11) | Gd2-O12 | 2.414 (11) | Gd3-O15 | 2.408 (11) | |
| Gd1-N11 | 2.449 (15) | Gd2-O15 | 2.388 (10) | Gd3-O21 | 2.404 (11) | |
| Gd1-O5 | 2.332 (14) | Gd2-N9 | 2.462(16) | Gd3-O14 | 2.383 (11) | |
| Gd1-O9 | 2.335 (11) | Gd2-O16 | 2.487 (12) | Gd3-O8 | 2.344 (11) | |
| Gd1-O12 | 2.395 (10) | Gd2-O9 | 2.325 (12) | Gd3-N21 | 2.494 (14) | |
| Gd4-O13 | 2.390 (12) | Gd5-O29 | 2.397 (12) | Gd6-O29 | 2.384 (10) | |
| Gd4-O14 | 2.398 (11) | Gd5-O30 | 2.575 (12) | Gd6-O19 | 2.350 (11) | |
| Gd4-O5 | 2.341 (12) | Gd5-O19 | 2.468 (10) | Gd6-O13 | 2.386 (11) | |
| Gd4-N19 | 2.432 (19) | Gd5-N17 | 2.45 (2) | Gd6-Cl4 | 2.697 (5) | |
| Gd4-O7 | 2.324 (14) | Gd5-O6 | 2.323 (12) | Gd6-O28 | 2.383 (11) | |
| Gd7-O27 | 2.563 (3) | Gd8-O12 | 2.358 (11) | Gd9-O3 | 2.327 (11) | |
| Gd7-O26 | 2.413 (10) | Gd8-O26 | 2.372 (10) | Gd9-O24 | 2.400 (11) | |
| Gd7-O28 | 2.389 (10) | Gd8-O16 | 2.360 (10) | Gd9-O25 | 2.465 (13) | |
| Gd7-N13 | 2.478 (16) | Gd8-O24 | 2.383 (10) | Gd9-O37 | 2.409(10) | |
| Gd7-O4 | 2.309 (11) | Gd8-O25 | 2.360 (11) | Gd9-N5 | 2.443 (16) | |
| Gd10-O2 | 2.329 (11) | Gd11-O1 | 2.306 (11) | Gd12-O15 | 2.376 (10) | |
| Gd10-O3 | 2.361 (10) | Gd11-O2 | 2.354 (11) | Gd12-O22 | 2.374 (10) | |
| Gd10-O16 | 2.396 (12) | Gd11-O23 | 2.392 (11) | Gd12-O23 | 2.371 (11) | |
| Gd10-O23 | 2.371 (12) | Gd11-O36 | 2.417 (11) | Gd12-O34 | 2.364(10) | |
| Gd10-O24 | 2.390 (10) | Gd11-N23 | 2.508 (14) | Gd12-O36 | 2.351 (11) | |
| Gd13-O33 | 2.590 (2) | Gd14-O21 | 2.457 (11) | Gd15-O14 | 2.400 (10) | |
| Gd13-O34 | 2.391 (11) | Gd14-O22 | 2.418 (10) | Gd15-O20 | 2.353 (11) | |
| Gd13-O35 | 2.299 (11) | Gd14-O32 | 2.414 (10) | Gd15-O21 | 2.339 (11) | |
| Gd13-O36 | 2.490 (10) | Gd14-O33 | 2.511 (3) | Gd15-O31 | 2.363 (11) | |
| Gd13-N1 | 2.501 (13) | Gd14-O34 | 2.433 (10) | Gd15-O32 | 2.377 (11) | |
| Gd16-O10 | 2.320 (11) | Gd16-O27 | 2.539 (3) | Gd16-O28 | 2.370 (10) | |
| Gd16-N15 | 2.432 (16) |  |  |  |  | |
| **Bond angles (°)** | | | | | | |
| O5-Gd1-O9 | 116.8 (5) | O8-Gd2-O11 | 72.2 (4) | O7-Gd3-O11 | | 71.6 (4) |
| O9-Gd1-O11 | 72.8 (4) | O9-Gd2-O11 | 71.5 (4) | O8-Gd3-O7 | | 115.9 (5) |
| O12-Gd1-O17 | 71.8 (3) | O12-Gd2-O11 | 72.0 (3) | O14-Gd3-O11 | | 72.8 (3) |
| O13-Gd1-O11 | 73.3 (4) | O15-Gd2-O12 | 77.7 (4) | O15-Gd3-O22 | | 70.2 (4) |
| O5-Gd1-O12 | 140.2 (4) | N9-Gd2-O16 | 79.7 (5) | O15-Gd3-N21 | | 132.8 (5) |
| O5-Gd4-O13 | 70.6 (4) | O6-Gd5-O19 | 80.1 (4) | O18-Gd6-O13 | | 70.4 (4) |
| O7-Gd4-O5 | 115.7 (6) | O20-Gd5-O30 | 141.9 (3) | O28-Gd6-O29 | | 80.8 (4) |
| O13-Gd4-O14 | 78.8 (4) | O29-Gd5-N17 | 132.7 (6) | O28-Gd6-O13 | | 80.5 (4) |
| O14-Gd4-N19 | 132.7 (6) | O40-Gd5-O19 | 113.7 (4) | O19-Gd6-O13 | | 73.7 (4) |
| O19-Gd4-O20 | 68.9 (4) | O40-Gd5-O20 | 72.1 (4) | O19-Gd6-O28 | | 144.5 (4) |
| O4-Gd7-O10 | 115.3 (4) | O12-Gd8-O16 | 73.0 (4) | O3-Gd9-O37 | | 144.2 (4) |
| O10-Gd7-O17 | 149.7 (4) | O12-Gd8-O17 | 73.3 (4) | O3-Gd9-N5 | | 69.6 (5) |
| O17-Gd7-O27 | 138.8 (3) | O17-Gd8-O16 | 119.9 (4) | O37-Gd9-O25 | | 68.6 (4) |
| O26-Gd7-N13 | 132.8 (5) | O25-Gd8-O24 | 71.9 (4) | O37-Gd9-N5 | | 85.4 (5) |
| O28-Gd7-O27 | 72.2 (3) | O26-Gd8-O24 | 81.0 (4) | N5-Gd9-O25 | | 78.1 (5) |
| O2-Gd10-O3 | 115.1 (5) | O1-Gd11-O2 | 117.0 (4) | O22-Gd12-O15 | | 71.8 (4) |
| O3-Gd10-O16 | 84.3 (4) | O2-Gd11-O23 | 71.0 (4) | O23-Gd12-O15 | | 81.3 (4) |
| O16-Gd10-N7 | 86.4 (6) | O23-Gd11-O36 | 70.3 (4) | O34-Gd12-O22 | | 73.1 (4) |
| O24-Gd10-O16 | 71.4 (4) | O36-Gd11-N23 | 89.3 (5) | O36-Gd12-O34 | | 73.6 (4) |
| N7-Gd10-O39 | 78.9 (5) | O23-Gd11-N23 | 148.7 (5) | O39-Gd12-O15 | | 74.4 (4) |
| O34-Gd13-O33 | 72.4 (3) | O22-Gd14-O21 | 69.2 (3) | O20-Gd15-O14 | | 72.3 (4) |
| O35-Gd13-O34 | 71.1 (4) | O22-Gd14-N3 | 86.4 (5) | O21-Gd15-O20 | | 118.1 (4) |
| O36-Gd13-N1 | 79.4 (6) | O32-Gd14-O21 | 70.0 (4) | O31-Gd15-O32 | | 73.2 (4) |
| N1-Gd13-O33 | 117.3 (6) | O34-Gd14-O33 | 73.1 (3) | O40-Gd15-O14 | | 81.7 (4) |
| O34-Gd13-N1 | 133.0 (5) | O35-Gd14-O21 | 147.1 (4) | O40-Gd15-O32 | | 80.5 (4) |
| O10-Gd16-O27 | 72.0 (3) | O28-Gd16-O27 | 72.9 (4) | O10-Gd16-N15 | | 69.8 (4) |
| O28-Gd16-N15 | 132.3 (5) |  |  |  | |  |
| Symmetry code: (i) ^1^-X,+Y,3/2-Z, | | | | | | |

# Table S4. *SHAPE* analysis of the Ho1 for cluster Ho_32_.

| **Label** | **Shape** | **Symmetry** | **Distortion(**^o^**)** |
| --- | --- | --- | --- |
| HP-7 | *D*_7h_ | Heptagon | 37.976 |
| HPY-7 | *C*_6v_ | Heptagonal pyramid | 17.141 |
| HBPY-7 | *D*_5h_ | Pentagonal bipyramid | 9.771 |
| COC-7 | *C*_3v_ | Capped octahedron | 0.649 |
| CYPR-7 | *C*_2v_ | Capped trigonal prism | 2.653 |
| JPBPY-7 | *D*_5h_ | Johnson pentagonal bipyramid J13 | 12.730 |
| JETPY-7 | *C*_3v_ | Johnson elongated triangular pyramid J7 | 20.462 |

# Table S5. *SHAPE* analysis of the Ho2 for cluster Ho_32_.

| **Label** | **Shape** | **Symmetry** | **Distortion(**^o^**)** |
| --- | --- | --- | --- |
| OP-8 | *D*_8h_ | Octagon | 29.407 |
| HPY-8 | *C*_7v_ | Heptagonalpyramid | 21.051 |
| HBPY-8 | *D*_6h_ | Hexagonal bipyramid | 16.197 |
| CU-8 | *O*_h_ | Cube | 10.544 |
| SAPR-8 | *D*_4d_ | Square antiprism | 0.723 |
| TDD-8 | *D*_2d_ | Triangular dodecahedron | 2.932 |
| JGBF-8 | *D*_2d_ | Johnson gyrobifastigium J26 | 14.217 |
| JETBPY-8 | *D*_3h_ | Johnsonelongatedtriangular bipyramid J14 | 27.523 |
| JBTPR-8 | *C*_2v_ | Biaugmented trigonal prism J50 | 2.517 |
| BTPR-8 | *C*_2v_ | Biaugmented trigonal prism | 2.690 |
| JSD-8 | *D*_2d_ | Snub diphenoid J84 | 4.812 |
| TT-8 | *T*_d_ | Triakis tetrahedron | 10.899 |
| ETBPY-8 | *D*_3h_ | Elongated trigonal bipyramid | 22.202 |


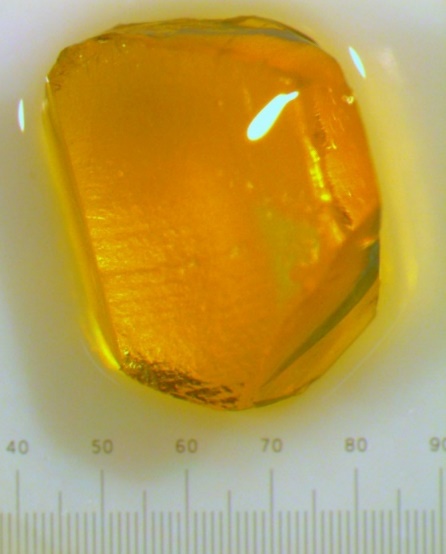


# Figure S1. The crystal photo of the spherical cluster Ho_32_.


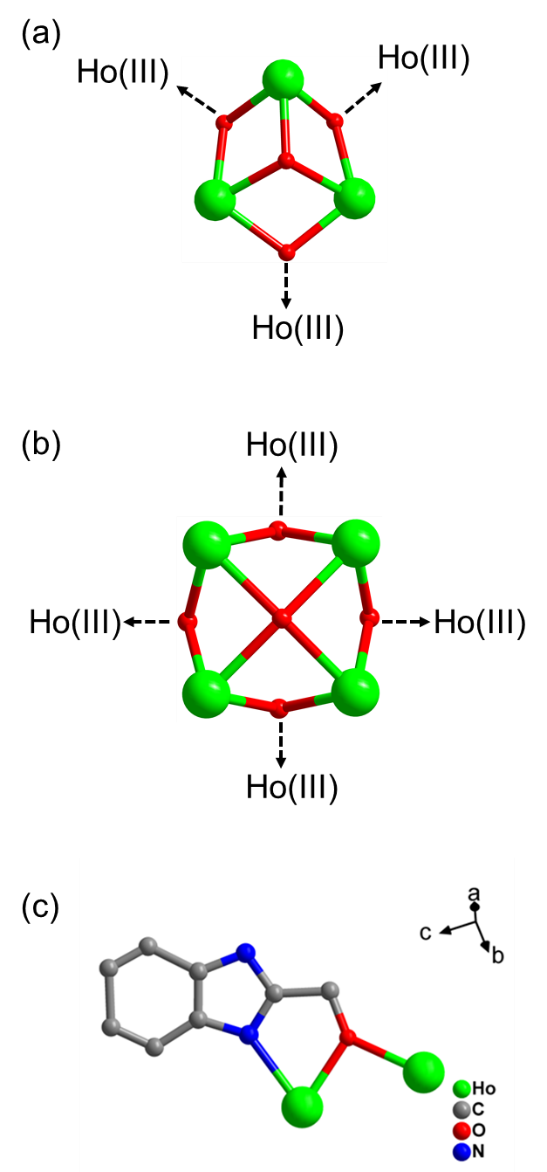


# Figure S2. Structure of trigonal {Ln_3_(*μ*_3_-OH)} (a) and square {Ln_4_(*μ*_4_-O)} (b); (c) Coordination mode of ligand L^-^.


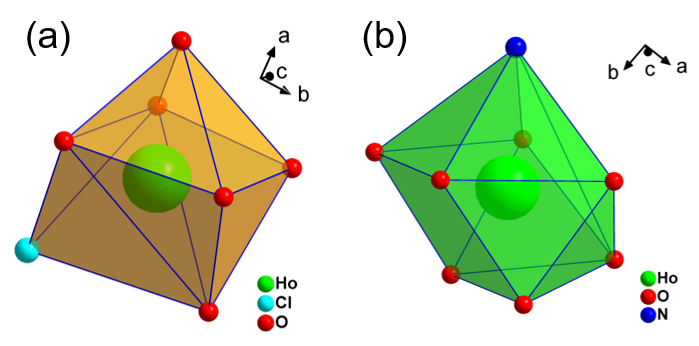


# Figure S3. Metal coordination configuration of Ho1 (a) and Ho2 (b).

The ligand L^-^ in the structure of the highly symmetrical spherical cluster **Ho_32_** has only one coordination mode: *μ*_3_-*ƞ*^1^:*ƞ*^2^ (Figure S2c). In addition, the metal center Ho1 is in the *C*_3v_ capped octahedron coordination environment formed by O_6_Cl (Table S4, Figure S3a), while Ho2 is in the *D*_4d_ square antiprism coordination environment formed by O_7_N (Table S5, Figure S3b).^4^


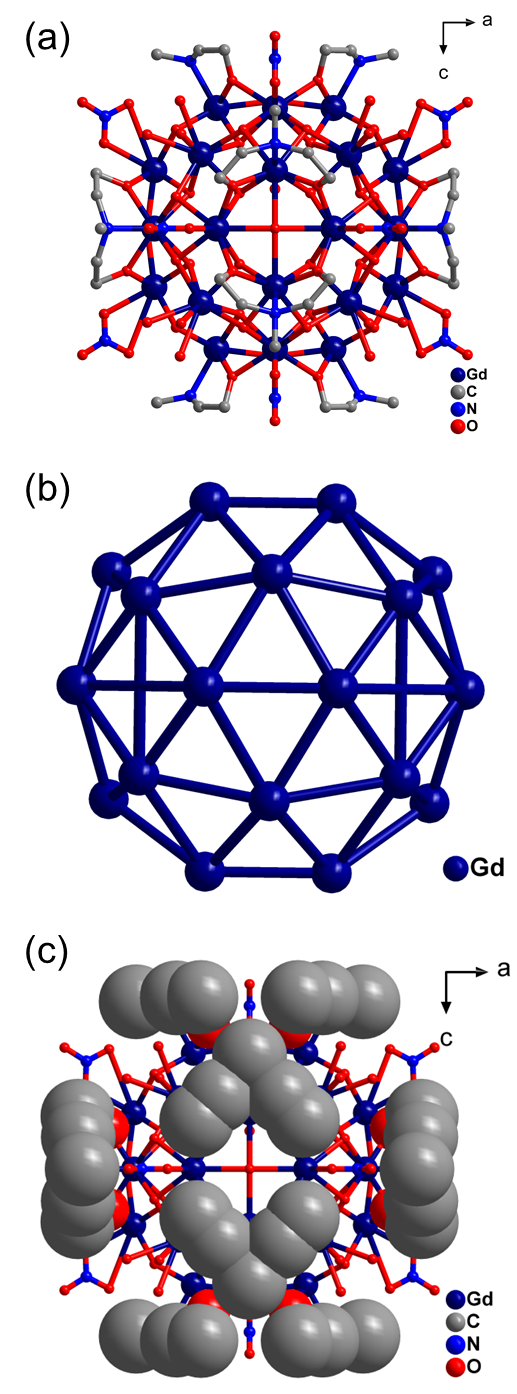


# Figure S4. Molecular structure (a), metal connection (b) and space-filling mode (c) of the cluster [Gd_32_(OH)_54_(mda)_12_(NO_3_)_12_(H_2_O)_24_⊃(H_2_O)_6_](OH)_6_ (for clarity, H atom, free OH^-^ ions and solvent molecules have been omitted).^5^

To date, high-nuclear lanthanide clusters have mainly been formed by hydrolysis and anion template methods, and have successfully expanded a series of different connection methods such as cages, wheels, hamburgers and ellipsoids. However, examples of spherical high-nuclear lanthanide clusters are still relatively rare. In 2017, Zheng *et al.* constructed a spherical gadolinium cluster [Gd_32_(OH)_54_(mda)_12_(NO_3_)_12_(H_2_O)_24_⊃(H_2_O)_6_](OH)_6_ (mdaH_2_ = *N*-methyl diethanolamine), but the cluster core of this spherical gadolinium cluster was not completely wrapped by the ligand mda, which led to its poor stability (Figure S4).^5^


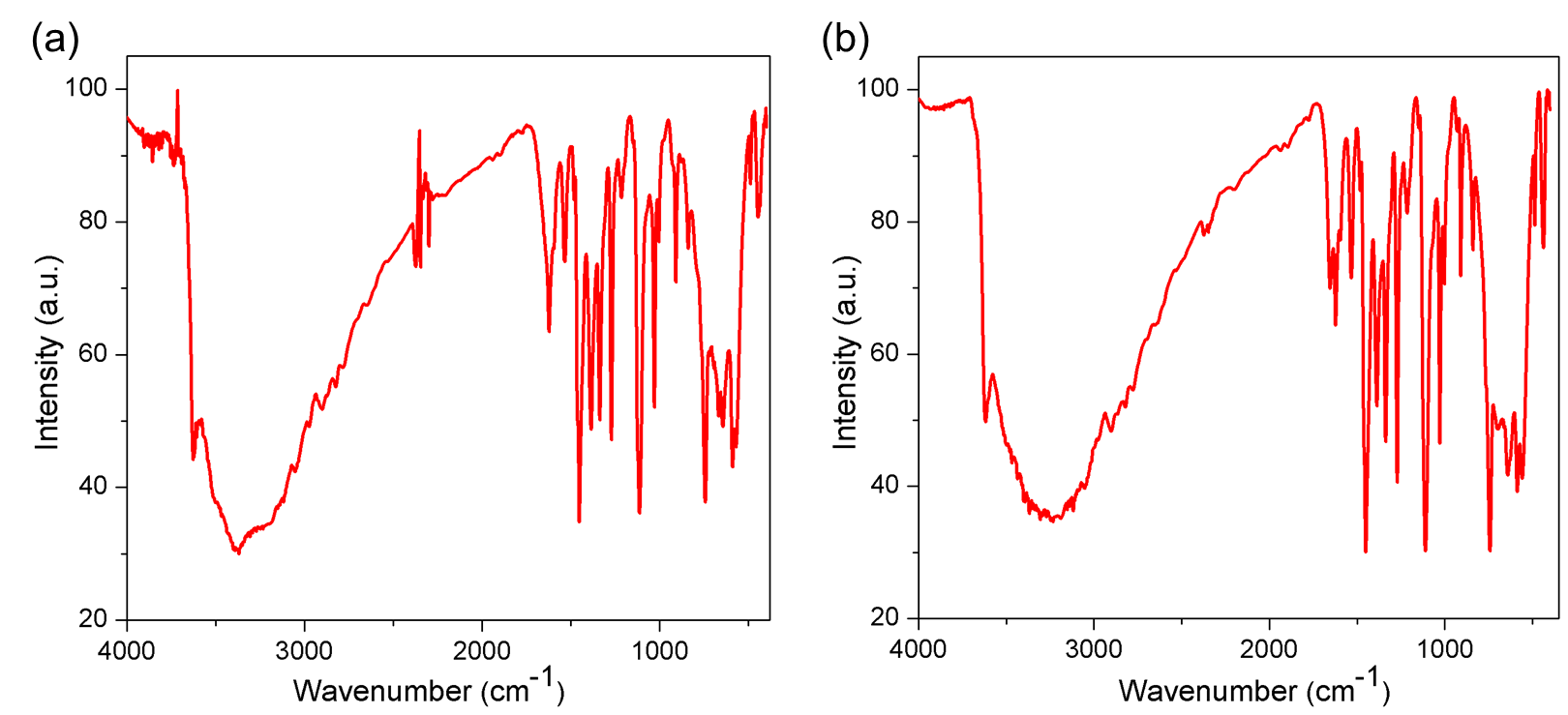


# Figure S5. Infrared spectra (IR) of clusters Ho_32_ (a) and Gd_32_ (b).


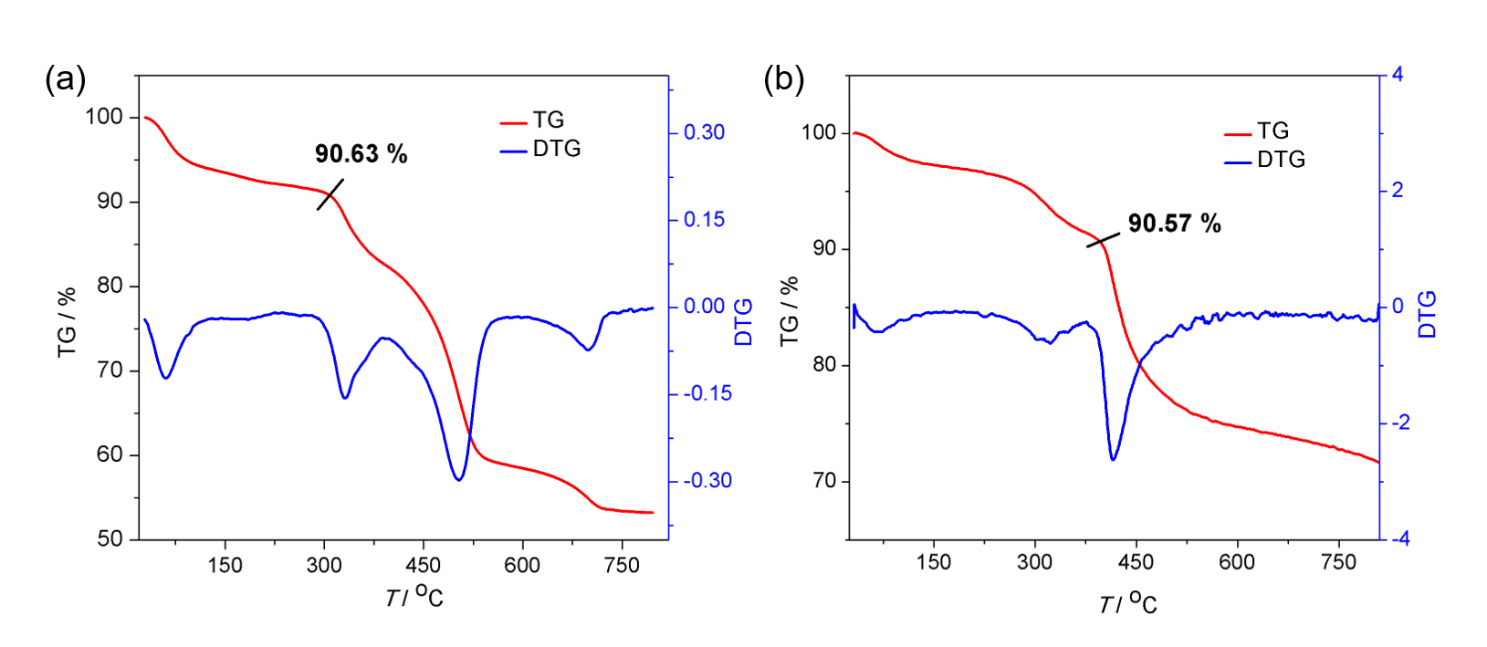


# Figure S6. TG curve of clusters Ho_32_ (a) and Gd_32_ (b).


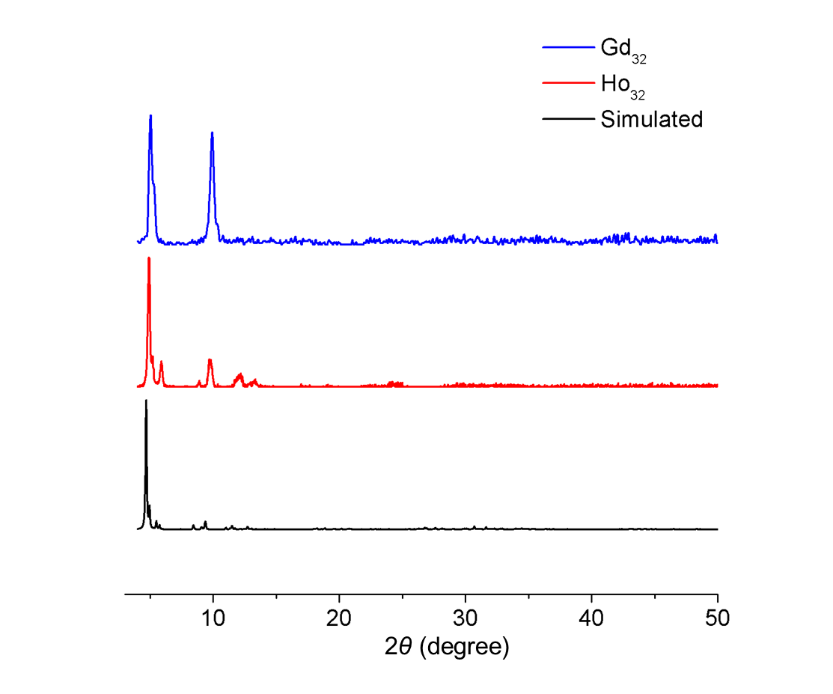


# Figure S7. Powder diffraction pattern (PXRD) of clusters Ho_32_ and Gd_32_.

Clusters **Ho_32_** and **Gd_32_** have similar infrared (IR, cm^-1^) absorption spectra, and the absorption peaks are located at 3419 (s, stretching vibration of *ν*(HO–H) in H_2_O molecules), 1624 (m, C=N stretching vibration of the imine group (–C=N–)), 1453 (s, C=C stretching vibrations in the aromatic ring), 1386 (m), 1338 (m), 1272 (s), 1114 (s, stretching vibration between the alcohol hydroxyl C−O in the ligand), 1030 (m), 744 (s), 645 (s) and 590 (s) (Figure S5). Thermogravimetric curve (TG) tests of **Ho_32_** and **Gd_32_** were carried out at 35-800 °C in a nitrogen atmosphere with a flow rate of 15 cm^3^/min at a heating rate of 10 °C/min. The weight loss of **Ho_32_** before 310 °C is approximately 9.37%, which corresponds to the loss of 45 free water molecules (7.23%), 5 methanol (1.43%) and 2 acetonitrile (0.73%) (Figure S6a). As the temperature rises above 535 °C, **Ho_32_** is rapidly decomposed. The weight loss of **Gd_32_** is approximately 9.43% before 410 °C, which corresponds to the loss of 35 free water molecules (5.8%), 6 methanol’s (0.30%) and 2 acetonitrile’s (0.38%) (Figure S6b). As the temperature further rises above 535 °C, **Gd_32_** is rapidly decomposes. The powder X-ray diffraction (PXRD) observations and simulation values for **Ho_32_** and **Gd_32_** were compared at room temperature, and the result indicated that all the phases are pure (Figure S7).

Since **Ho_32_** and **Gd_32_** are isomorphic, we only used **Ho_32_** to examine the stability under different conditions. On this basis, we further used HRESI-MS with different ion source voltages to test the solution behaviour of **Ho_32_** under bombardment conditions with different ion source energies. We dissolved a small amount of **Ho_32_** crystals in chromatographically pure DMF and used anhydrous methanol, which is prone to vaporization to dilute the crystals for HRESI-MS testing (Figure 2a).


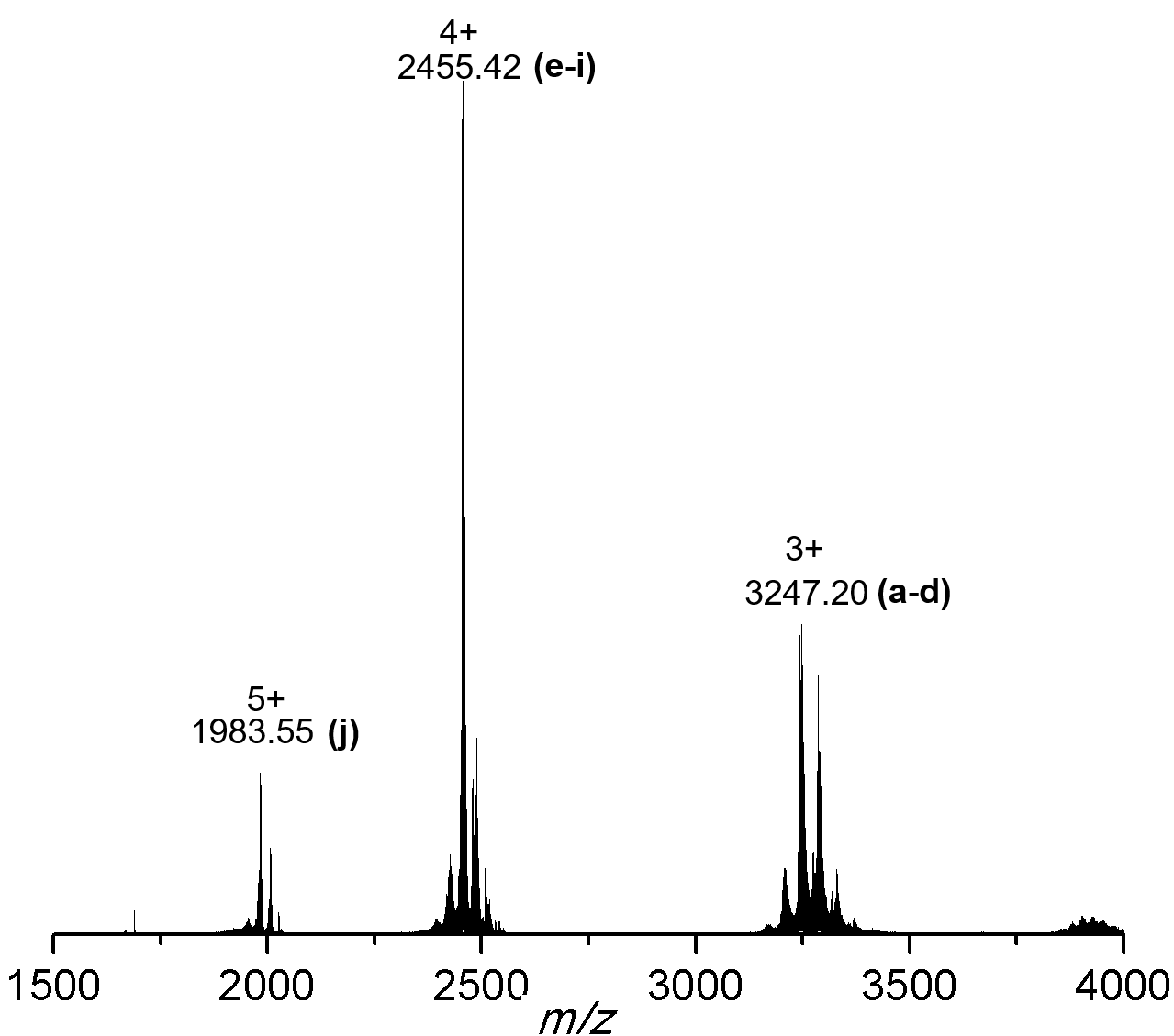


# Figure S8. Positive HRESI-MS spectra of Ho_32_ in DMF.

# Table S6. Major species assigned in the HRESI-MS of Ho_32_ in positive mode.

| Fragments | Exp. *m/z* | Calc. *m/z* |
| --- | --- | --- |
| (a) [Ho_32_(L)_21_(OH)_48_(O)_6_(Cl)_13_(H^+^)]^3+^ | 3247.20 | 3247.21 |
| (b) [Ho_32_(L)_20_(OH)_48_(O)_6_(Cl)_13_(CH_3_OH)_2_(H_2_O)(CH_3_CN)_2_]^3+^ | 3252.54 | 3252.56 |
| (c) [Ho_32_(L)_20_(OH)_48_(O)_6_(Cl)_13_(CH_3_OH)_2_(H_2_O)_4_]^3+^ | 3242.54 | 3242.55 |
| (d) [Ho_32_(L)_21_(OH)_48_(O)_6_(Cl)_12_(CH_3_CN)_2_(H_2_O)_4_]^3+^ | 3285.89 | 3285.91 |
| (e) [Ho_32_(L)_21_(OH)_48_(O)_6_(Cl)_13_(H^+^)_2_(H_2_O)(CH_3_OH)_2_]^4+^ | 2455.42 | 2455.43 |
| (f) [Ho_32_(L)_22_(OH)_48_(O)_6_(Cl)_13_(H^+^)_3_(CH_3_OH)_2_]^4+^ | 2488.43 | 2488.44 |
| (g) [Ho_32_(L)_21_(OH)_48_(O)_6_(Cl)_13_(H^+^)_2_(CH_3_CN)_2_(CH_3_OH)_2_(H_2_O)_3_]^4+^ | 2485.69 | 2485.69 |
| (h) [Ho_32_(L)_21_(OH)_48_(O)_6_(Cl)_13_(H^+^)_2_(CH_3_CN)_3_(CH_3_OH)(H_2_O)]^4+^ | 2479.18 | 2479.11 |
| (i) [Ho_32_(L)_21_(OH)_48_(O)_6_(Cl)_13_(H^+^)_2_(CH_3_CN)(CH_3_OH)(H_2_O)]^4+^ | 2458.68 | 2458.68 |
| (j) [Ho_32_(L)_22_(OH)_48_(O)_6_(Cl)_11_(H^+^)_2_(H_2_O)(CH_3_CN)_2_]^5+^ | 1983.35 | 1983.36 |


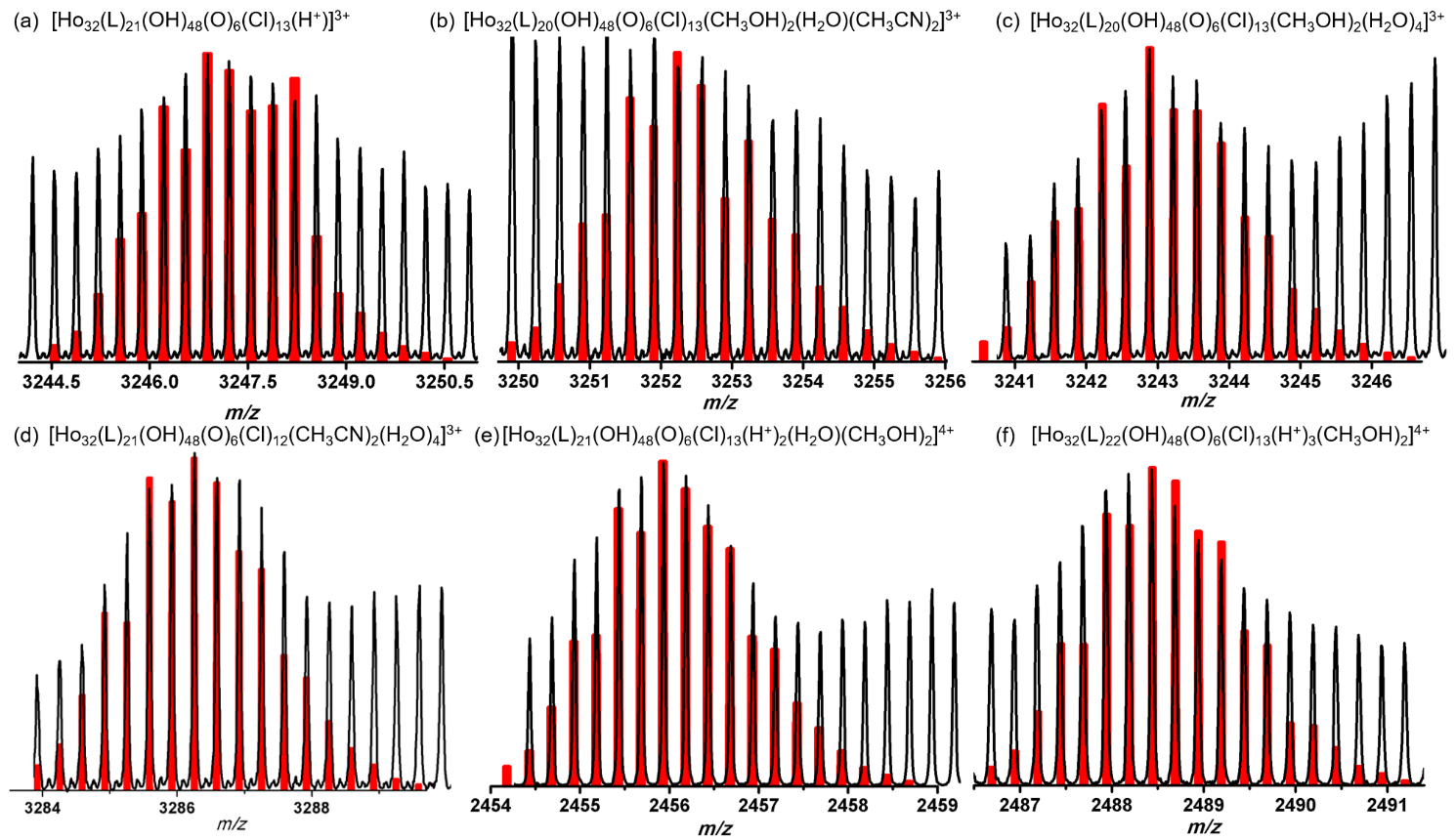


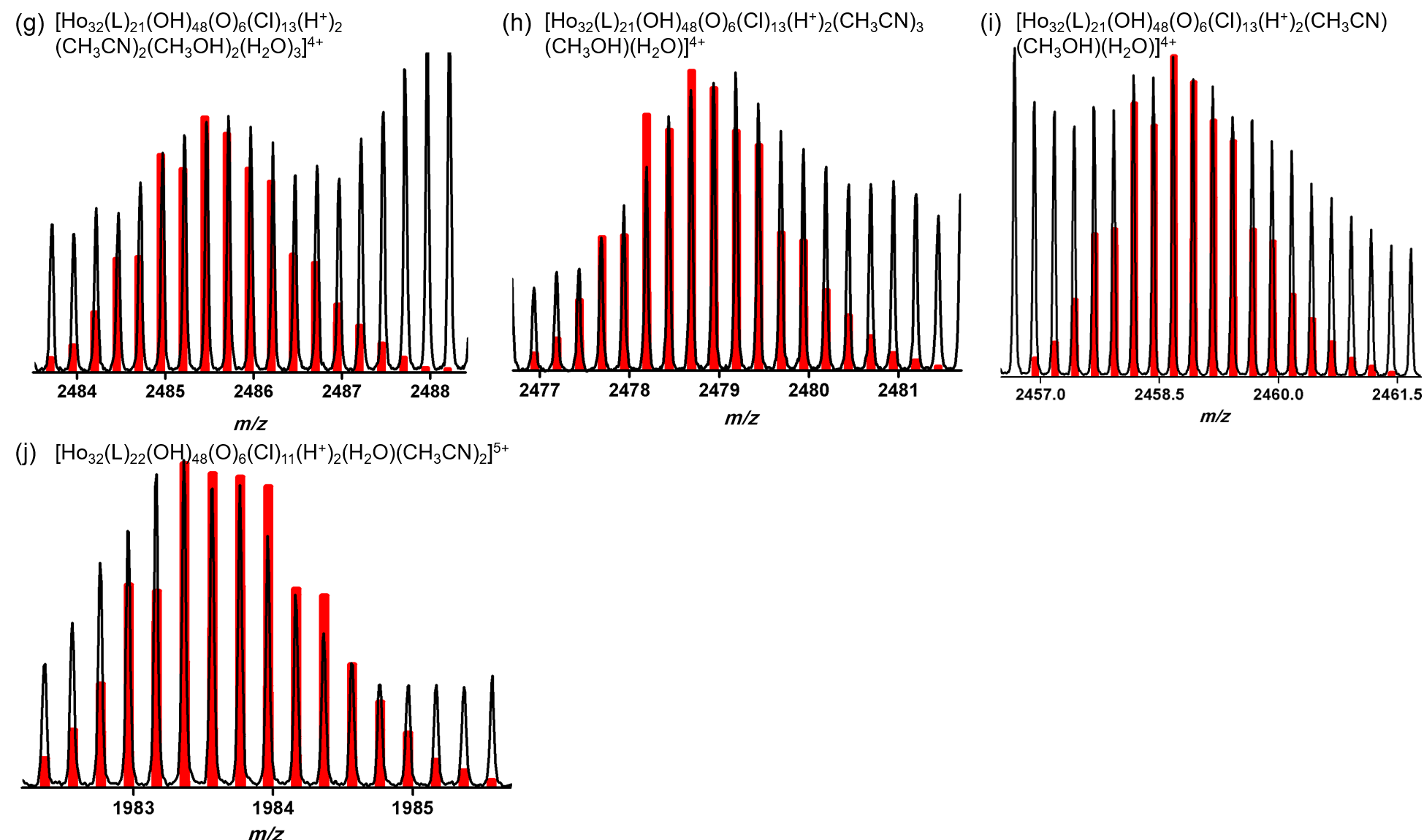


# Figure S9. The superposed simulated and observed spectra of several species for cluster Ho_32_.

# Table S7. Major species assigned in the HRESI-MS of Ho_32_ with different in-source CID (0-65 eV) in positive mode.

| Fragments | Exp. *m/z* | Calc. *m/z* |
| --- | --- | --- |
| [Ho_32_(L)_22_(OH)_48_(O)_6_(Cl)_11_(H^+^)_2_(H_2_O)]^5+^ | 1983.35 | 1983.36 |
| [Ho_32_(L)_21_(OH)_48_(O)_6_(Cl)_13_(H^+^)_2_(CH_3_CN)(CH_3_OH)(H_2_O)]^4+^ | 2458.68 | 2458.68 |
| [Ho_32_(L)_22_(OH)_48_(O)_6_(Cl)_13_(H^+^)_3_(CH_3_OH)_2_]^4+^ | 2488.43 | 2488.44 |
| [Ho_32_(L)_21_(OH)_48_(O)_6_(Cl)_13_(H^+^)_2_(CH_3_CN)_2_(CH_3_OH)_2_(H_2_O)_3_]^4+^ | 2485.69 | 2485.69 |
| [Ho_32_(L)_21_(OH)_48_(O)_6_(Cl)_13_(H^+^)_2_(H_2_O)(CH_3_OH)_2_]^4+^ | 2455.42 | 2455.43 |
| [Ho_32_(L)_21_(OH)_48_(O)_6_(Cl)_13_(H^+^)_2_(CH_3_CN)_3_(CH_3_OH)(H_2_O)]^4+^ | 2478.69 | 2478.69 |
| [Ho_32_(L)_23_(OH)_48_(O)_6_(Cl)_9_(DMF)(CH_3_CN)_2_]^4+^ | 2511.71 | 2511.50 |
| [Ho_32_(L)_22_(OH)_48_(O)_6_(Cl)_11_(CH_3_CN)]^3+^ | 3286.23 | 3286.59 |
| [Ho_32_(L)_24_(OH)_48_(O)_6_(Cl)_10_(H)(CH_3_CN)]^3+^ | 3372.58 | 3372.31 |


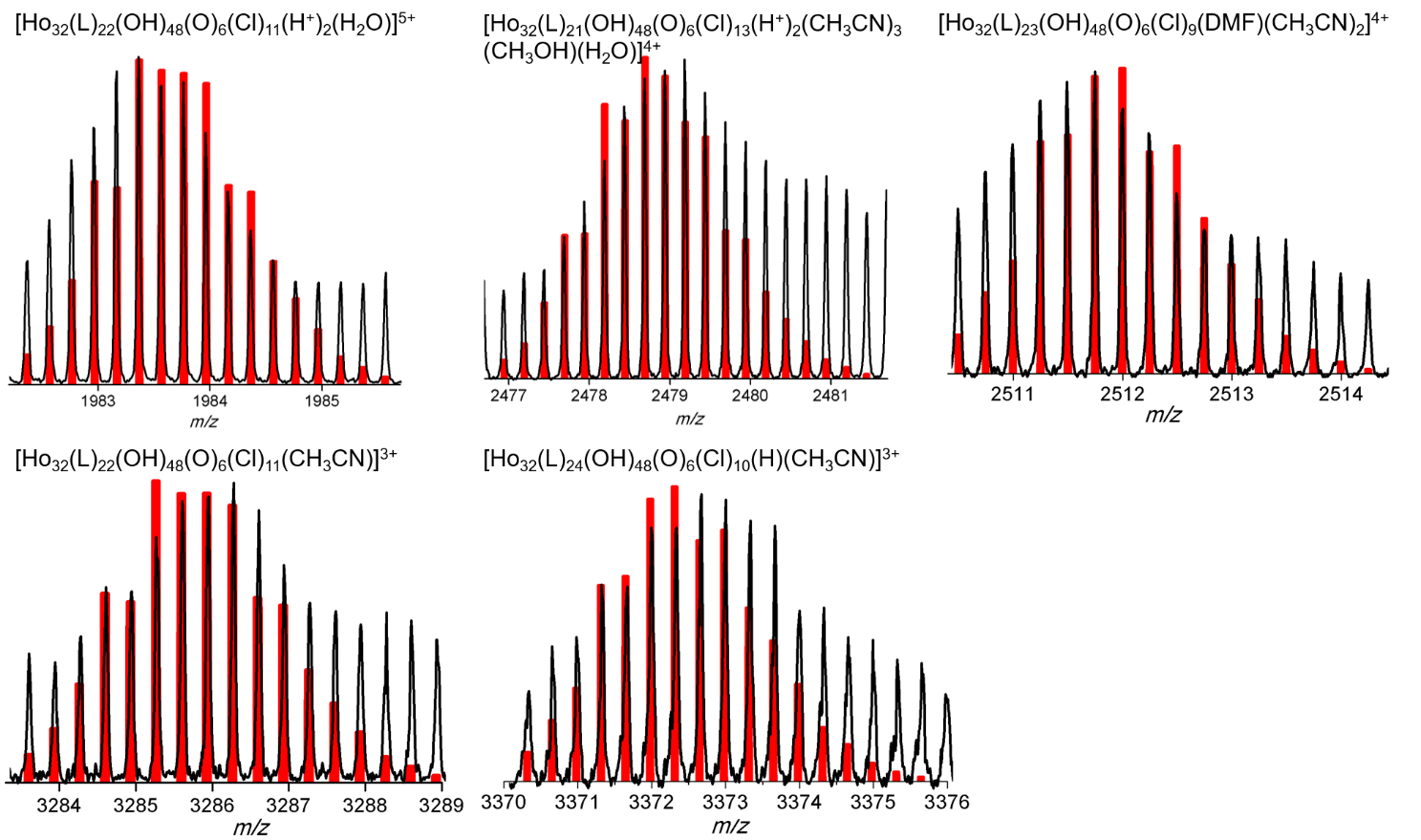


# Figure S10. The superposed simulated and observed spectra of several species for Ho_32_ with different in-source CID (0-65 eV).

# Table S8. Major species assigned in the HRESI-MS of Gd_32_ in positive mode.

| Fragments | Exp. *m/z* | | Calc. *m/z* |
| --- | --- | --- | --- |
| (a) [Gd_32_(L)_22_(OH)_48_(O)_6_(Cl)_12_(DMF)(H_2_O)_3_(H^+^)]^3+^ | 3245.16 | 3245.11 | |
| (b) [Gd_32_(L)_21_(OH)_48_(O)_6_(Cl)_13_(H^+^)(CH_3_CN)(H_2_O)]^3+^ | 3203.15 | 3203.19 | |
| (c) [Gd_32_(L)_19_(OH)_48_(O)_6_(Cl)_15_(CH_3_OH)(H_2_O)_2_(H^+^)(DMF)_2_]^3+^ | 3162.46 | 3162.44 | |
| (d) [Gd_32_(L)_19_(OH)_48_(O)_6_(Cl)_15_(CH_3_CN)(H_2_O)_3_(H^+^)]^3+^ | 3122.77 | 3122.72 | |
| (e) [Gd_32_(L)_23_(OH)_48_(O)_6_(Cl)_8_(H_2_O)_4_(OH^-^)]^4+^ | 2425.38 | 2425.39 | |
| (f) [Gd_32_(L)_21_(OH)_48_(O)_6_(Cl)_13_(H_2_O)_2_(CH_3_CN)(H^+^)]^4+^ | 2393.37 | 2393.37 | |
| (g) [Gd_32_(L)_19_(OH)_48_(O)_6_(Cl)_13_(DMF)_2_(CH_3_CN)_2_(H_2_O)]^4+^ | 2361.85 | 2361.86 | |
| (h) [Gd_32_(L)_22_(OH)_48_(O)_6_(Cl)_11_(H^+^)_2_(CH_3_OH)_2_(H_2_O)]^5+^ | 1931.11 | 1931.16 | |
| (i) [Gd_32_(L)_20_(OH)_48_(O)_6_(Cl)_11_(CH_3_CN)_4_(CH_3_OH)(H_2_O)_3_]^5+^ | 1905.49 | 1905.54 | |


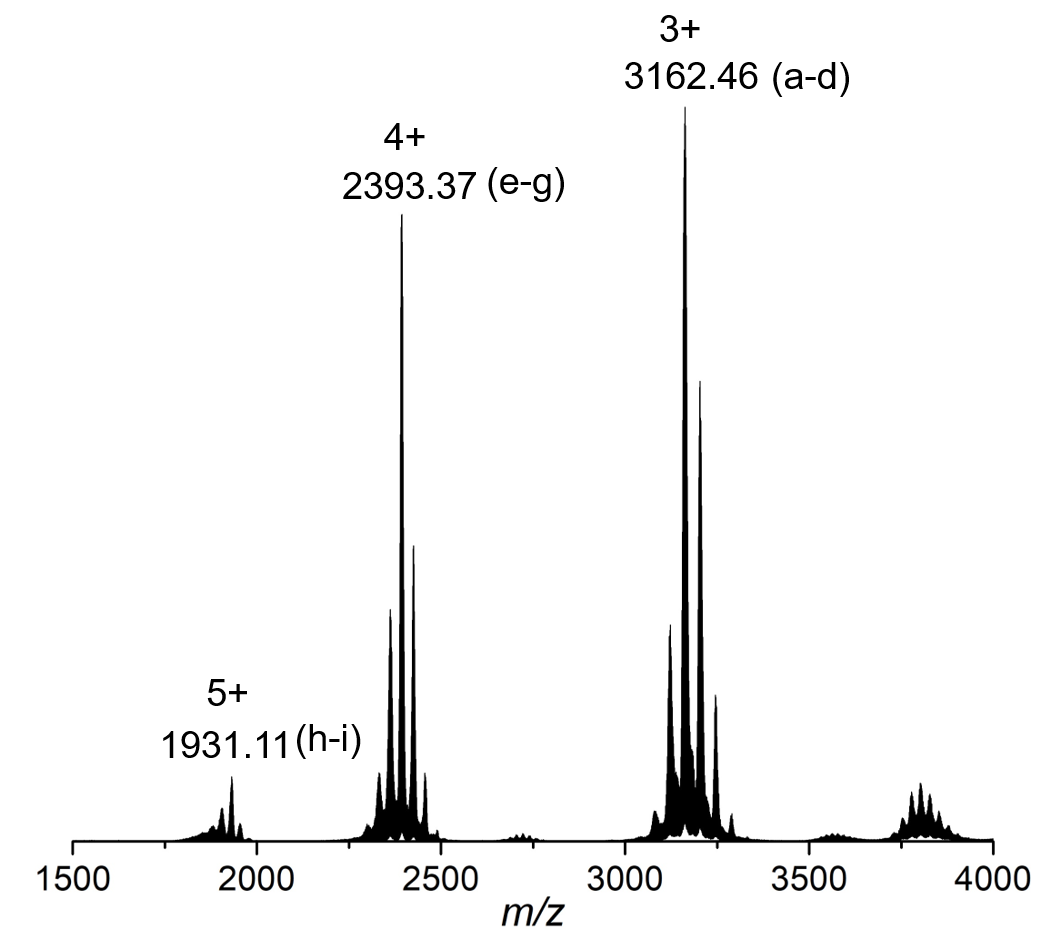


# Figure S11. Positive HRESI-MS spectra of Gd_32_ in DMF.


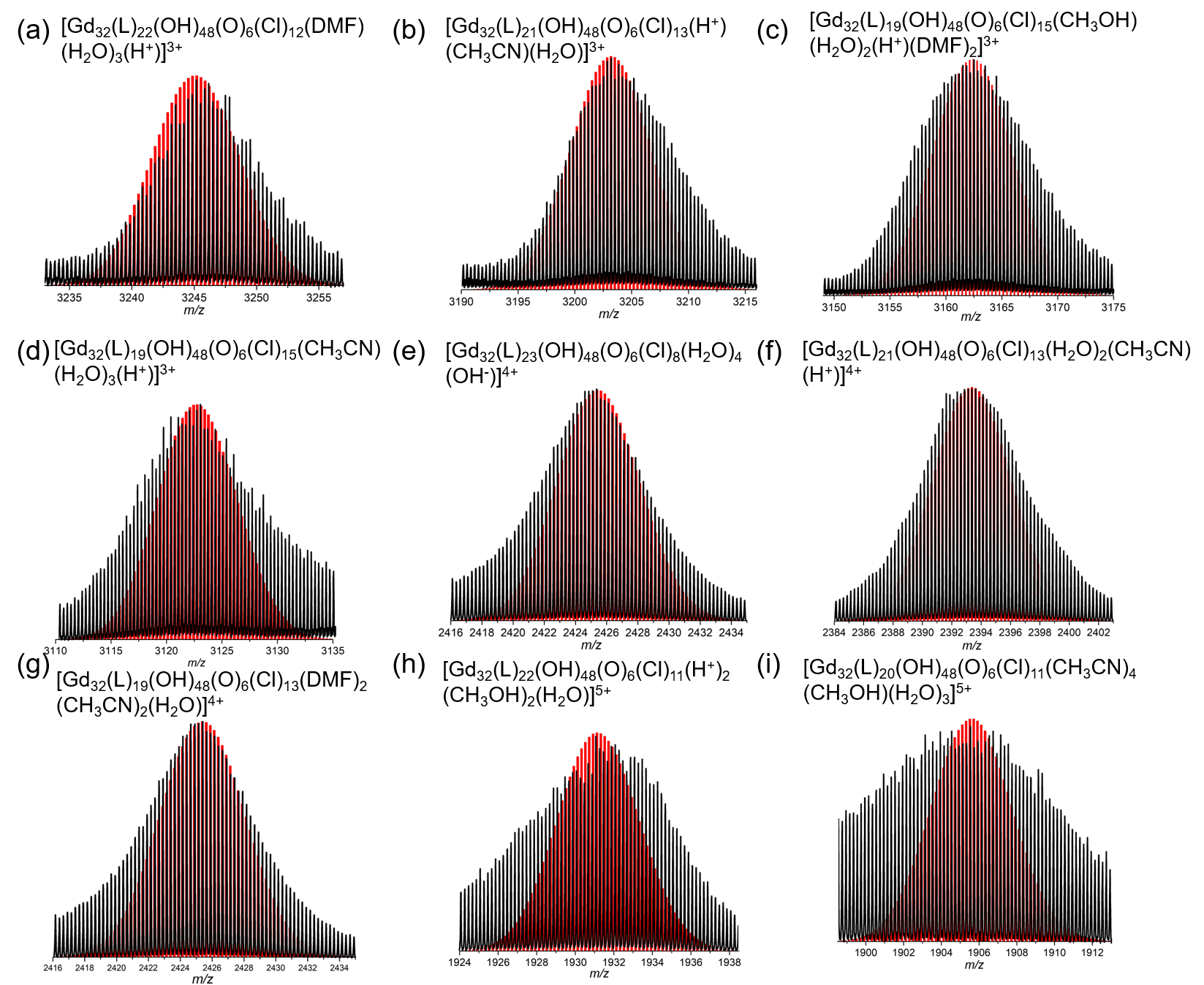


# Figure S12. The superposed simulated and observed spectra of several species for cluster Gd_32_.


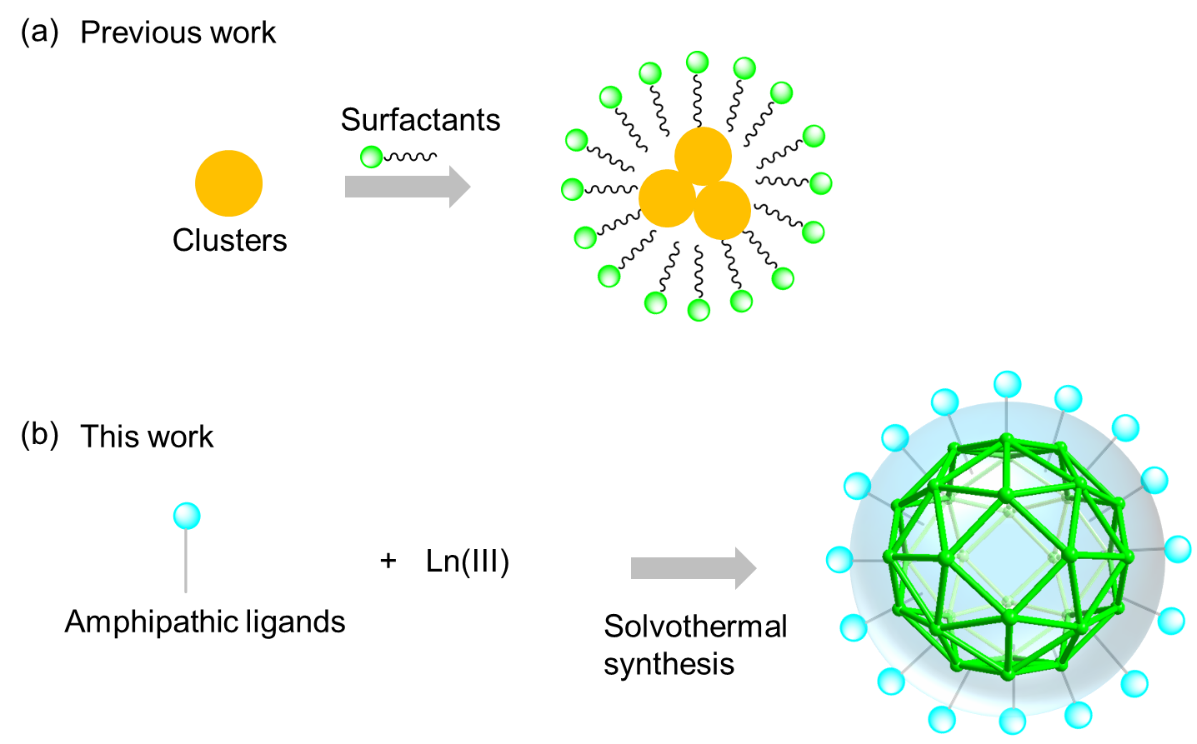


# Figure S13. (a) Schematic diagram of surfactant-coated high-nuclear clusters;^6,7^ (b) The flow chart of using amphiphilic ligands to synthesize high-nuclear rare earth clusters with high water stability and water solubility.

To date, most of the high-nuclear lanthanide clusters are formed by small-volume anions connected to the metal centre, and a large-scale exposure of the cluster cores cause them having low stabilities in solutions (especially aqueous solutions). Recently, of using amphiphilic polymers or SiO_2_ to wrap high-nuclear clusters to form nanospheres has gradually emerged to promote the water solubility and water stability of the clusters (Figure S13a).^6,7^ Because it is difficult to determine the loading of the high-nucleus clusters inside the nanospheres during the wrapping process, the uniformity and stability still cannot be greatly improved. High-nuclear clusters constructed by using amphiphilic ligands and the ligands gather on the periphery of the cluster core, which is equivalent to covering each cluster molecule with a dense protective layer and can greatly improve its water solubility, water stability and uniformity (Figure S13b).

# Table S9. Major species assigned in the time-dependent HRESI-MS for tracked the formation of Ho_32_ in positive mode.

| ***m/z*** | **Fragments** | **Relative Intensity** | | | | | | | | | | | |
| --- | --- | --- | --- | --- | --- | --- | --- | --- | --- | --- | --- | --- | --- |
|  |  | 0 min | 10min | 20min | 30min | 45min | 1 h | 2h | 3h | 4h | 5h | 12h | 24h |
| 650.00 | [Ho_3_(L)_3_(Cl)_2_(H_2_O)_3_(CH_3_CN)_5_(OH)_2_]^2+^ (cal.649.53) | 0.68 | 0.98 | 0.62 | 0.58 | 0.54 | 0.54 | 0.11 | 0.02 | 0 | 0 | 0 | 0 |
| 813.95 | [Ho_4_(L)_5_(Cl)_3_(O)(OH)_2_(CH_3_CN)(H_2_O)_2_(H^+^)_2_]^2+^ (cal.814.49) | 0.34 | 0.14 | 0.14 | 0.02 | 0 | 0 | 0 | 0 | 0 | 0 | 0 | 0 |
| 1174.95 | [Ho_6_(L)_7_(OH)_6_(Cl)_4_(CH_3_OH)(H_2_O)_3_]^2+^ (cal.1174.46) | 0.19 | 0.09 | 0.16 | 0.04 | 0.02 | 0.02 | 0 | 0 | 0 | 0 | 0 | 0 |
| 1178.93 | [Ho_6_(L)_6_(OH)_4_(Cl)_7_(CH_3_CN)(CH_3_OH)_3_(H_2_O)_2_]^2+^ (cal.1179.92) | 0.23 | 0.18 | 0.26 | 0.14 | 0.11 | 0.15 | 0 | 0 | 0 | 0 | 0 | 0 |
| 1225.98 | [Ho_6_(L)_7_(Cl)_4_(OH)_5_(DMF)(CH_3_CN)_2_(CH_3_OH)(H_2_O)]^2+^ (cal.1225.50) | 0.09 | 0.04 | 0.09 | 0.03 | 0 | 0 | 0 | 0 | 0 | 0 | 0 | 0 |
| 1231.01 | [Ho_6_(L)_7_(OH)_6_(Cl)_3_(CH_3_CN)(DMF)(H_2_O)_5_(CH_3_OH)]^2+^ (cal.1232.03) | 0.17 | 0.10 | 0.20 | 0.10 | 0.06 | 0.09 | 0 | 0 | 0 | 0 | 0 | 0 |
| 1336.96 | [Ho_3_(L)_3_(OH)_2_(Cl)_4_(H_2_O)_2_(CH_3_CN)_3_(CH_3_OH)_2_]^+^ (cal.1336.00) | 1.00 | 1.00 | 1.00 | 1.00 | 1.00 | 1.00 | 0.12 | 0.03 | 0 | 0 | 0 | 0 |
| 1372.90 | [Ho_3_(L)_4_(Cl)_4_(CH_3_OH)(H_2_O)_2_(CH_3_CN)_2_]^+^ (cal.1372.99) | 0.19 | 0.36 | 0.38 | 0.41 | 0.44 | 0.49 | 0.07 | 0 | 0 | 0 | 0 | 0 |
| 1397.94 | [Ho_3_(L)_4_(Cl)_6_(H^+^)_3_(CH_3_OH)_2_(H_2_O)_2_]^+^ (cal.1397.92) | 0.22 | 0.17 | 0.17 | 0.19 | 0.18 | 0.20 | 0 | 0 | 0 | 0 | 0 | 0 |
| 1502.20 | [Ho_16_(L)_19_(O)_11_(Cl)_5_(CH_3_CN)(DMF)_2_(CH_3_OH)]^4+^ (cal.1502.47) | 0 | 0 | 0 | 0 | 0.13 | 0.24 | 0.32 | 0.28 | 0.29 | 0.20 | 0.05 | 0.02 |
| 1664.91 | [Ho_4_(L)_4_(O)(Cl)_4_(OH)(CH_3_CN)_3_(CH_3_OH)_2_(H_2_O)_3_(H^+^)]^+^ (cal.1664.99) | 0.51 | 0.10 | 0.17 | 0.03 | 0 | 0 | 0 | 0 | 0 | 0 | 0 | 0 |
| 1690.49 | [Ho_28_(L)_15_(OH)_48_(O)_5_(Cl)_8_(HH)(DMF)_3_(H_2_O)_2_(CH_3_CN)_3_(CH_3_OH)_2_]^5+^ (cal.1690.21) | 0 | 0 | 0 | 0 | 0 | 0 | 0 | 0.16 | 0.17 | 0.18 | 0.15 | 0.16 |
| 1980.24 | [Ho_16_(L)_19_(OH)_16_(O)_3_(Cl)_4_(DMF)_3_(H_2_O)_2_]^3+^(cal.1980.39) | 0 | 0 | 0 | 0 | 0.38 | 0.69 | 0.99 | 0.99 | 0.90 | 0.77 | 0.16 | 0 |
| 2014.26 | [Ho_16_(L)_19_(OH)_15_(O)_4_(Cl)_5_(CH_3_CN)_2_(CH_3_OH)]^3+^(cal.2014.63) | 0 | 0 | 0 | 0 | 0.38 | 0.63 | 0.98 | 0.84 | 0.99 | 0.70 | 0.13 | 0.11 |
| 2444.74 | [Ho_32_(L)_21_(OH)_48_(O)_6_(Cl)_13_(H^+^)_2_(H_2_O)_2_]^4+^(cal.2444.42) | 0 | 0 | 0 | 0 | 0 | 0.06 | 0.90 | 0.53 | 0.30 | 0.52 | 0.10 | 0.01 |
| 2520.69 | [Ho_32_(L)_24_(OH)_48_(O)_6_(Cl)_8_(CH_3_CN)(H_2_O)_2_]^4+^(cal.2520.25) | 0 | 0 | 0 | 0 | 0 | 0 | 0.40 | 1.00 | 1.00 | 1.00 | 0.87 | 1.00 |
| 2553.22 | [Ho_32_(L)_24_(OH)_48_(O)_6_(Cl)_9_(CH_3_CN)_2_(DMF)(OH)]^4+^(cal.2553.01) | 0 | 0 | 0 | 0 | 0 | 0 | 0.28 | 0.75 | 0.83 | 0.94 | 1.00 | 1.00 |
| 3271.33 | [Ho_32_(L)_21_(OH)_48_(O)_6_(Cl)_11_(CH_3_OH)(H_2_O)_2_(H^+^)_2_(DMF)]^3+^(cal.3271.27) | 0 | 0 | 0 | 0 | 0 | 0 | 0.36 | 0.28 | 0.16 | 0.25 | 0 | 0 |
| 3339.30 | [Ho_32_(L)_23_(OH)_48_(O)_6_(Cl)_11_(H_2_O)_3_]^3+^(cal.3339.28) | 0 | 0 | 0 | 0 | 0 | 0 | 0.20 | 0.17 | 0.29 | 0.25 | 0.13 | 0.14 |
| 3372.60 | [Ho_32_(L)_23_(OH)_48_(O)_6_(Cl)_10_(DMF)(CH_3_CN)_2_(OH)_2_(H^+^)]^3+^(cal.3372.65) | 0 | 0 | 0 | 0 | 0 | 0 | 0.16 | 0.51 | 0.55 | 0.48 | 0.50 | 0.59 |
| 3415.94 | [Ho_32_(L)_24_(OH)_48_(O)_6_(Cl)_10_(DMF)(CH_3_CN)_2_(H_2_O)]^3+^(cal.3416.00) | 0 | 0 | 0 | 0 | 0 | 0 | 0.12 | 0.38 | 0.45 | 0.44 | 0.52 | 0.57 |


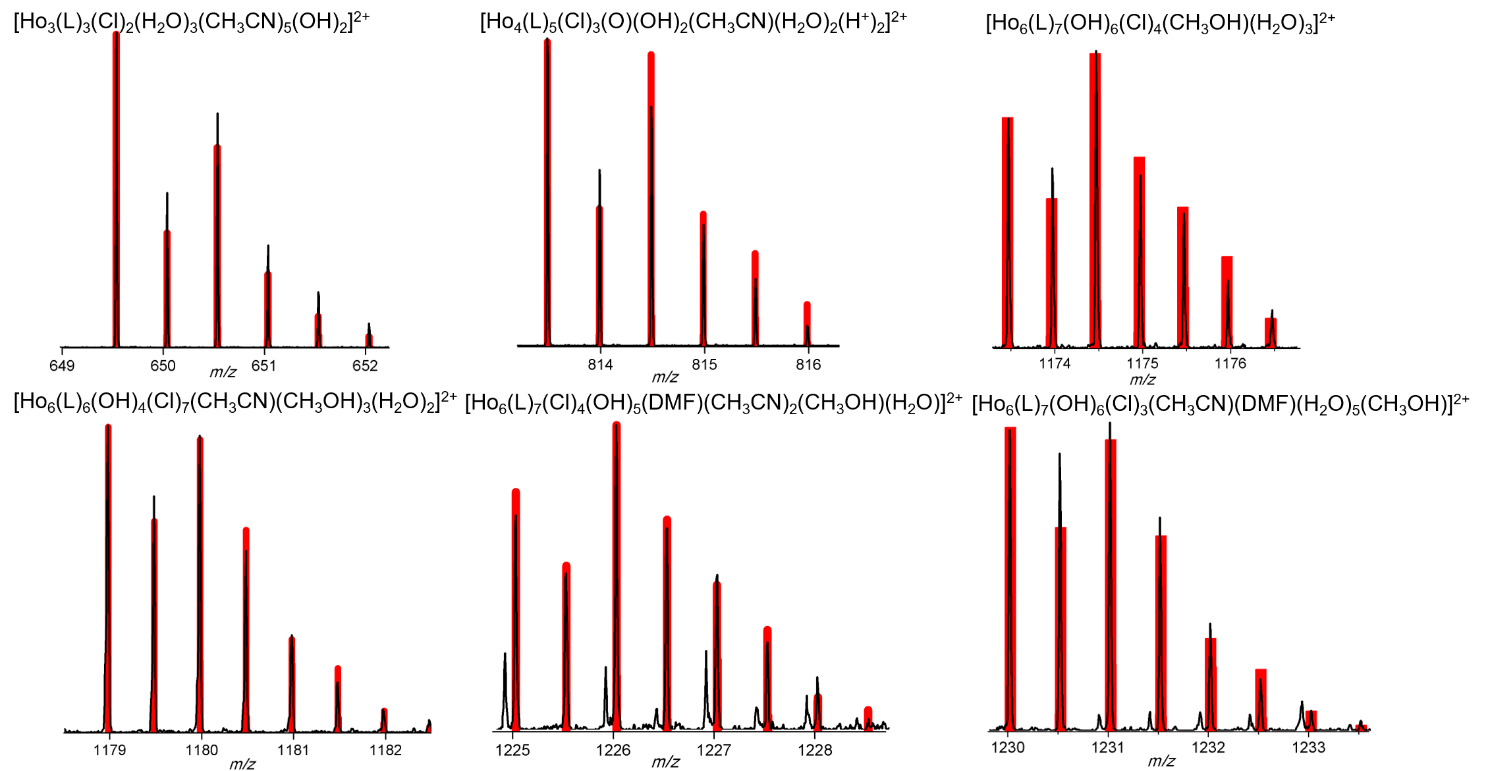


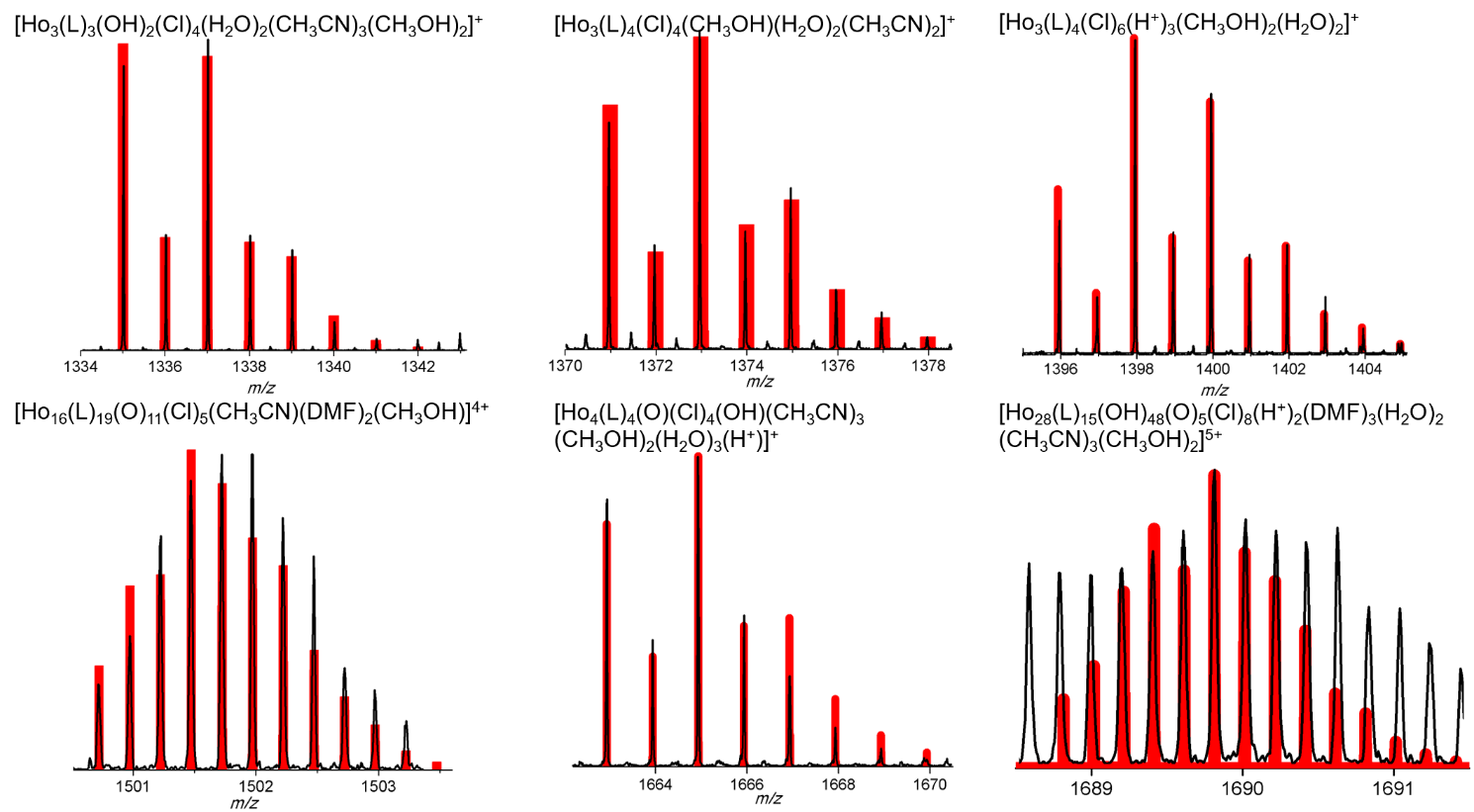


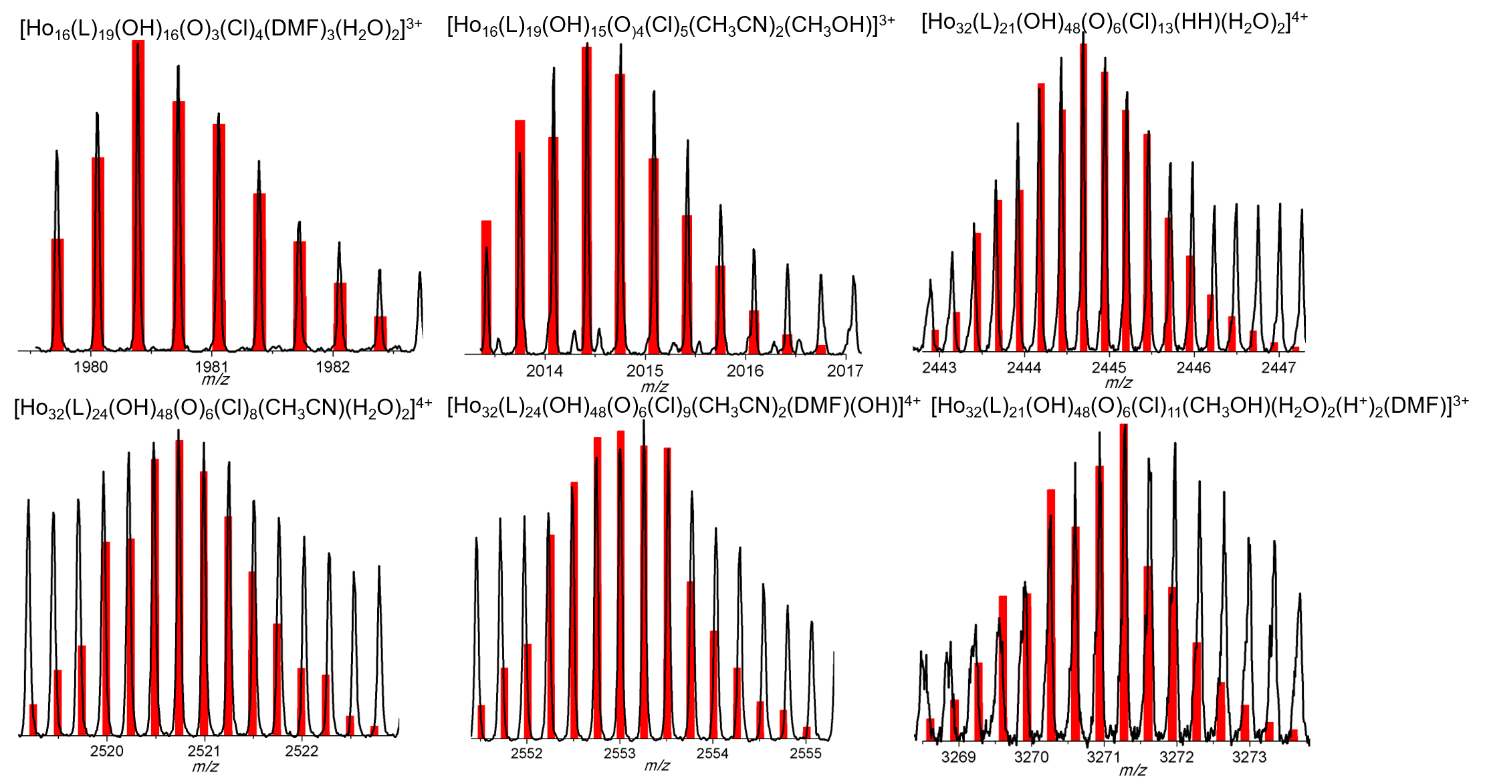


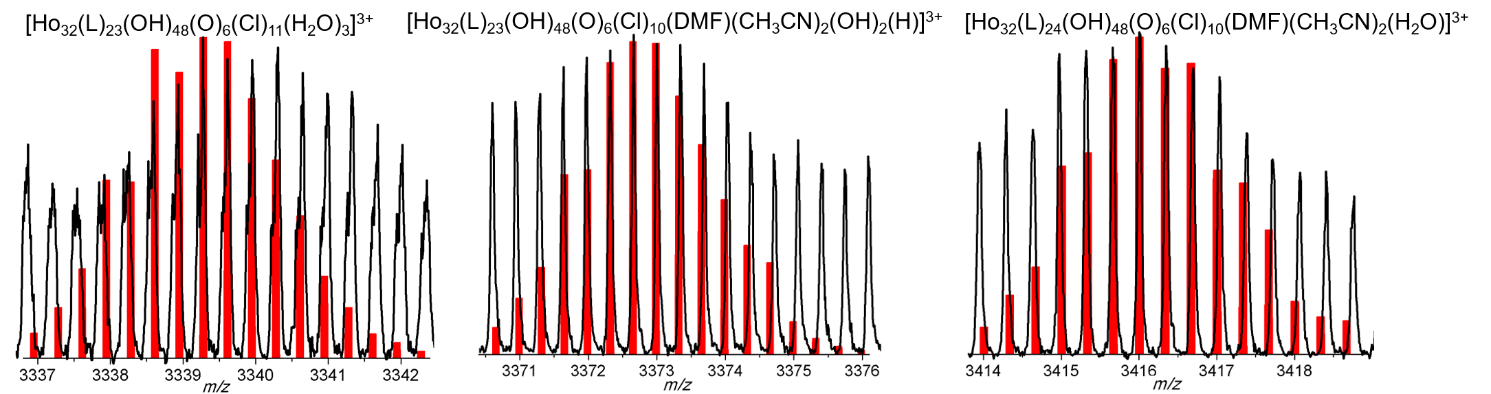


# Figure S14. The time-dependent HRESI-MS superposed simulated and observed spectra of several species for tracked the formation of Ho_32_.

Tracking the formation process of high-nuclear clusters and explorating their self-assembly mechanism have always attracted much attention.^8-10^ In 2022, Zheng *et al.* designed and separated a series of high-nucleus lanthanide clusters with twelve nuclei, thirty-four nuclei, forty-eight nuclei and sixty nuclei through the joint action of various template anions, and revealed the self-assembly process of cage-shaped sixty nuclei lanthanide cluster.^11^ The excellent stability of spherical cluster **Ho_32_** has encouraged studies to be performed regarding its formation process and self-assembly mechanism. Multiple sets of parallel experiments were performed under the same conditions, and the same amount of reaction solution was removed from the reaction system at specific time intervals. The HRESI-MS test was performed immediately after each equal amount of the above reaction solution was diluted in the same proportion. HRESI-MS was used to quickly detect the types of molecular ion peaks and their abundance changes in the reaction solution at different time periods, to speculate the most likely self-assembly mechanism of the high-nuclear spherical cluster **Ho_32_** (Figures 3a, 3b and S14, Table S9). In the initial stage of the reaction (0 min), the molecular ion peaks in the reaction system are mainly Ho_3_(L)_3_ ([Ho_3_(L)_3_(Cl)_2_(H_2_O)_3_(CH_3_CN)_5_(OH)_2_]^2+^, *m/z* = 650 and [Ho_3_(L)_3_(OH)_2_(Cl)_4_(H_2_O)_2_(CH_3_CN)_3_(CH_3_OH)_2_]^+^, *m/z* = 1336.96), Ho_3_(L)_4_ ([Ho_3_(L)_4_(Cl)_4_(CH_3_OH)(H_2_O)_2_(CH_3_CN)_2_]^+^, *m/z* = 1372.90 and [Ho_3_(L)_4_(Cl)_6_(H^+^)_3_(CH_3_OH)_2_(H_2_O)_2_]^+^, *m/z* = 1397.94) and Ho_4_(L)_4_ ([Ho_4_(L)_4_(O)(Cl)_4_(OH)(CH_3_CN)_3_(CH_3_OH)_2_(H_2_O)_3_(H^+^)]^+^, *m/z* = 1664.91). In addition, a small number of molecular ion peaks in Ho_6_(L)_6_ ([Ho_6_(L)_6_(OH)_4_(Cl)_7_(CH_3_CN)(CH_3_OH)_3_(H_2_O)_2_]^2+^, *m/z* = 1178.93) and Ho_6_(L)_7_ ([Ho_6_(L)_7_(OH)_6_(Cl)_4_(CH_3_OH)(H_2_O)_3_]^2+^, *m/z* = 1174.95 and [Ho_6_(L)_7_(Cl)_4_(OH)_5_(DMF)(CH_3_CN)_2_(CH_3_OH)(H_2_O)]^2+^ *m/z* = 1225.98) appeared at the same time. As the reaction progressed to 10 min, the fragment intensity of Ho_3_(L)_3_ and Ho_3_(L)_4_ in the reaction system reached the strongest value. However, the intensity of the Ho_4_(L)_4_ fragment rapidly weakened, indicating that the process of Ho_4_(L)_4_ binding to Ho(III) ions to produce Ho_6_(L)_4_ is relatively rapid. When the reaction reached 20 min, the abundance of Ho_3_(L)_3_ decreases slightly, while the abundance of Ho_6_(L)_6_ increases to a certain extent. Until the reaction progressed to 30 min, molecular ion peaks related to Ho_16_(L)_19_ ([Ho_16_(L)_19_(O)_11_(Cl)_5_(CH_3_CN)(DMF)_2_(CH_3_OH)]^4+^, *m/z* = 1502.20; [Ho_16_(L)_19_(OH)_16_(O)_3_(Cl)_4_(DMF)_3_(H_2_O)_2_]^3+^, *m/z* = 1980.24 and [Ho_16_(L)_19_(OH)_15_(O_)4_(Cl)_5_(CH_3_CN)_2_(CH_3_OH)]^3+^ *m/z* = 2014.26) appeared in the reaction solution. The reaction continued for 1 h, and the intensity of Ho_16_(L)_19_ gradually increased due to the continuous accumulation of Ho_16_(L)_19_ in the reaction system. As the reaction progressed to 2 h, a molecular ion peak of Ho_28_(L)_15_ ([Ho_28_(L)_15_(OH)_48_(O)_5_(Cl)_8_(H^+^)_2_(DMF)_3_(H_2_O)_2_(CH_3_CN)_3_(CH_3_OH)_2_]^5+^, *m/z* = 1690.49) appeared in the reaction solution. At the same time, the molecular ion peak of the final product Ho_32_(L)_21_ ([Ho_32_(L)_21_(OH)_48_(O)_6_(Cl)_13_(H^+^)_2_(H_2_O)_2_]^4+^, *m/z* = 2444.74 and [Ho_32_(L)_21_(OH)_48_(O)_6_(Cl)_11_(CH_3_OH)(H_2_O)_2_(H^+^)_2_(DMF)]^3+^, *m/z* = 3271.33); Ho_32_(L)_23_ ([Ho_32_(L)_23_(OH)_48_(O)_6_(Cl)_11_(H_2_O)_3_]^3+^, *m/z* = 3339.30 and [Ho_32_(L)_23_(OH)_48_(O)_6_(Cl)_10_(DMF)(CH_3_CN)_2_(OH)_2_(H^+^)]^3+^, *m/z* = 3372.60) also appeared in the reaction solution (Figures 3a and S14, Table S9). In addition, the molecular ion peaks related to Ho_3_(L)_3_, Ho_3_(L)_4_, Ho_4_(L)_4_, Ho_4_(L)_5_, Ho_6_(L)_6_, and Ho_6_(L)_7_ in the reaction solution disappeared. As the reaction proceeds further, the molecular ion peaks related to Ho_16_(L)_19_ and Ho_28_(L)_15_ in the reaction solution gradually weaken, and the molecular ion peaks related to the spherical clusters Ho_32_(L)_24_/Ho_32_(L)_21_/Ho_32_(L)_23_ gradually increase. It is worth noting that the intensity of the molecular ion peaks related to Ho_4_(L)_4_, Ho_6_(L)_6_ and Ho_28_(L)_20_ was low during the formation of the spherical cluster **Ho_32_**, indicating that the Ho_4_(L)_4_/Ho_4_(L)_5_ → Ho_6_(L)_6_/Ho_6_(L)_7_ → Ho_16_(L)_19_ and Ho_28_(L)_15_ → Ho_32_(L)_24_/Ho_32_(L)_21_/Ho_32_(L)_23_ processes were very fast. Therefore, the rate-determining steps (RDS) in the formation of spherical clusters **Ho_32_** are Ho_3_(L)_3_/Ho_3_(L)_4_ → Ho_4_(L)_4_/Ho_4_(L)_5_ and Ho_16_(L)_19_ → Ho_28_(L)_15_ (Figure 3c).

The benzimidazole-2-methanol, the end-group-coordinated Cl^-^ and the bridged OH^-^ in the **Gd_32_** structure can all efficiently provide a large number of hydrogen-bond-donor/acceptor groups. The huge cavity and strong hydrogen bonding with water molecules may lead to the ultra-high *T*_1_ relaxivity of **Gd_32_**, and provide a special microenvironment around the metal centres between inner-sphere and second-sphere (Figure S15).^12-15^


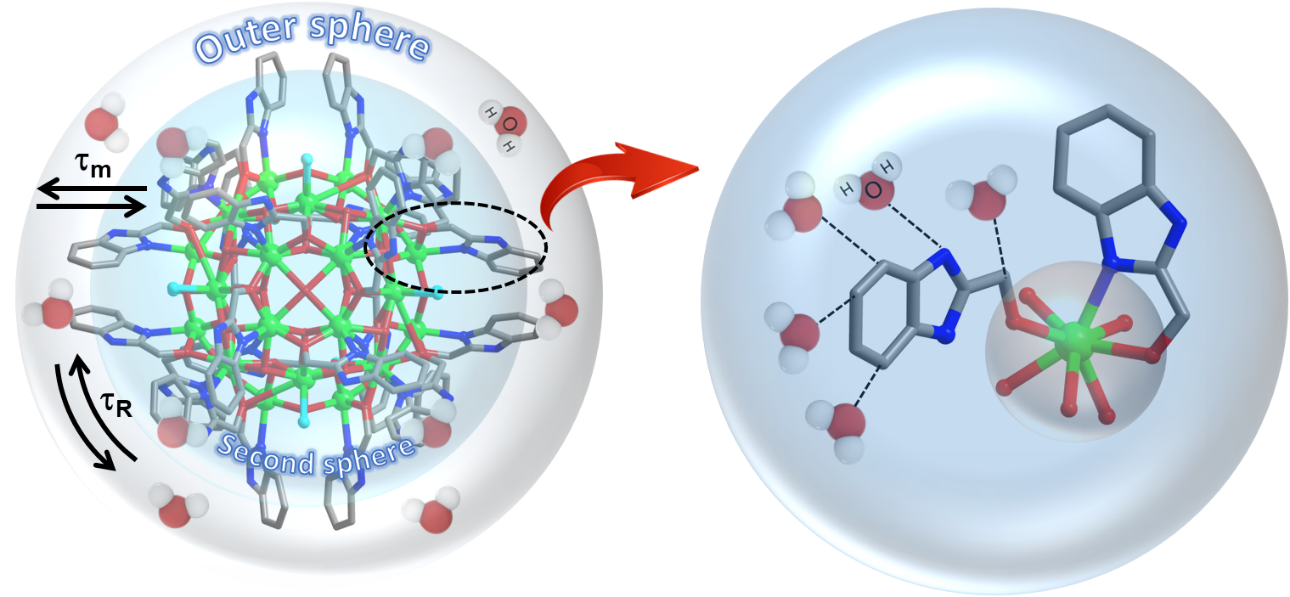


# Figure S15. Pictorial description of the parameters that influence the relaxivity of Gd_32_ (H atom, free Cl^-^ ions and solvent molecules have been omitted for clarity).

^1^H NMRD was performed on 0.25 Tesla Bench-top Fast Field Cycling NMR Relaxometer (SMARtracer™) and the results show that the longitudinal relaxivity of **Gd_32_** (23 μM) under different magnetic field strengths is stable around 535 mM^-1^s^-1^ when the proton Larmor frequency is less than 1 MHz (Figure S16). As the proton Larmor frequency increases to 10 MHz, the longitudinal relaxivity of **Gd_32_** drops rapidly to 113 mM^-1^s^-1^. According to the Solomon-Bloembergen-Morgan (SBM) theory, several factors affecting the longitudinal relaxation of **Gd_32_** were optimized and calculated to explain the main reason for its high longitudinal relaxivity (Table S10).^16,17^ First, the huge molecular weight and highly rigid cluster structure can effectively bind the Gd(III) ions inside the structure, which can significantly improve the overall turnover time (*τ*_R_) of the **Gd_32_**.^16,18^ Compared with the reported Gd-DTPA (*τ*_R_ = 58 ps),^19,20^ the τ_R_ of **Gd_32_** (*τ*_R_ = 95000 ps) is increased by about 1638-fold. Second, the spherical **Gd_32_** cluster have very high symmetry, which can significantly reduce the zero-field splitting (ZFS) energy (Δ^2^) of **Gd_32_**. Compared with Gd-DTPA (Δ^2^ = 0.46 × 10^20^ s^-2^), the Δ^2^ of **Gd_32_** (Δ^2^ = 0.2 × 10^16^ s^-2^) is greatly reduced. In addition, the internal water binding time *τ*_m_ of **Gd_32_** (*τ*_m_ = 5 ns) was also significantly reduced compared with that of Gd-DTPA (*τ*_m_ = 142 ns).^21^ Finally, the presence of surfactant-like ligands surrounding the cluster core Gd(III) center and the large cavity inside the spherical cluster can significantly reduce the external water diffusion characteristic time (*τ*_D_) of **Gd_32_**. Compared to Gd-DTPA (*τ*_D_ = 44 ps), the *τ*_D_ of **Gd_32_** (*τ*_D_ = 1.95 ps) is reduced by a factor of 22.6. The amphiphilic ligands and large cavity in the **Gd_32_** structure provide confinement space for the exchange of H_2_O between the inside and outside, resulting in the reduction of *τ*_m_ and *τ*_D_. All in all, the combined effect of the above-mentioned influencing factors leads to **Gd_32_** with excellent *T*_1_ relaxivity.


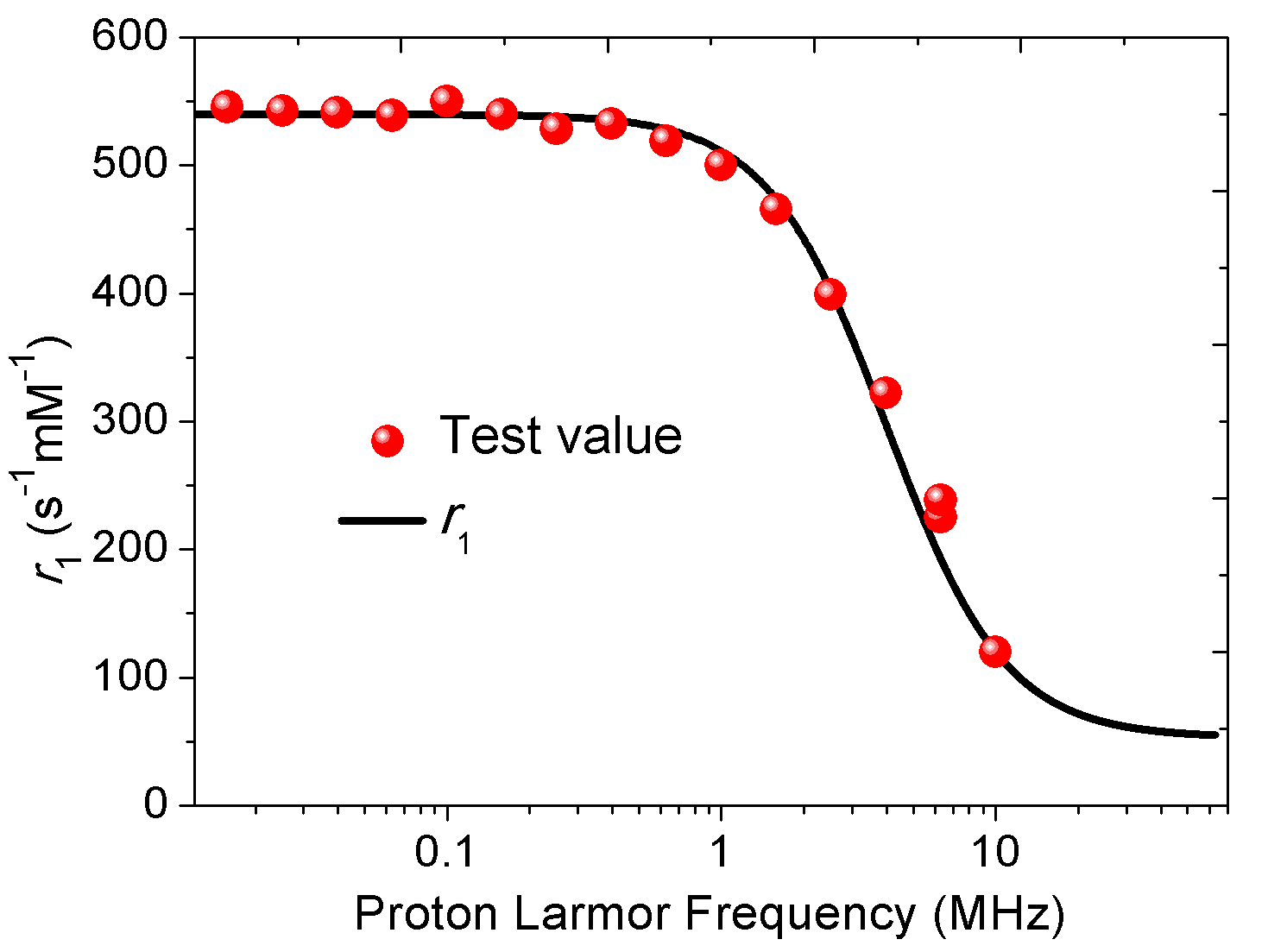


# Figure S16. Longitudinal relaxivity distributions of cluster Gd_32_ under different field strengths (different hydrogen proton Larmor frequencies), and fitting curves based on Solomon-Bloembergen Morgan (SBM) paramagnetic relaxation theory.

# Table S10. Parameters during longitudinal relaxation of Gd-DTPA and Gd_32_.

|  | **Gd-DTPA**^19,20^ | **Gd_32_** |
| --- | --- | --- |
| *τ*_R_ (ps) | 58 | 95000 |
| *τ*_V_ (ps) | 25 | 26 |
| *τ*_m_ (ns) | 142 | 5 |
| *Δ*^2^ (s^-2^) | 0.46 × 10^20^ | 0.2 × 10^16^ |
| *τ*_D_ (ps) | 44 | 1.95 |


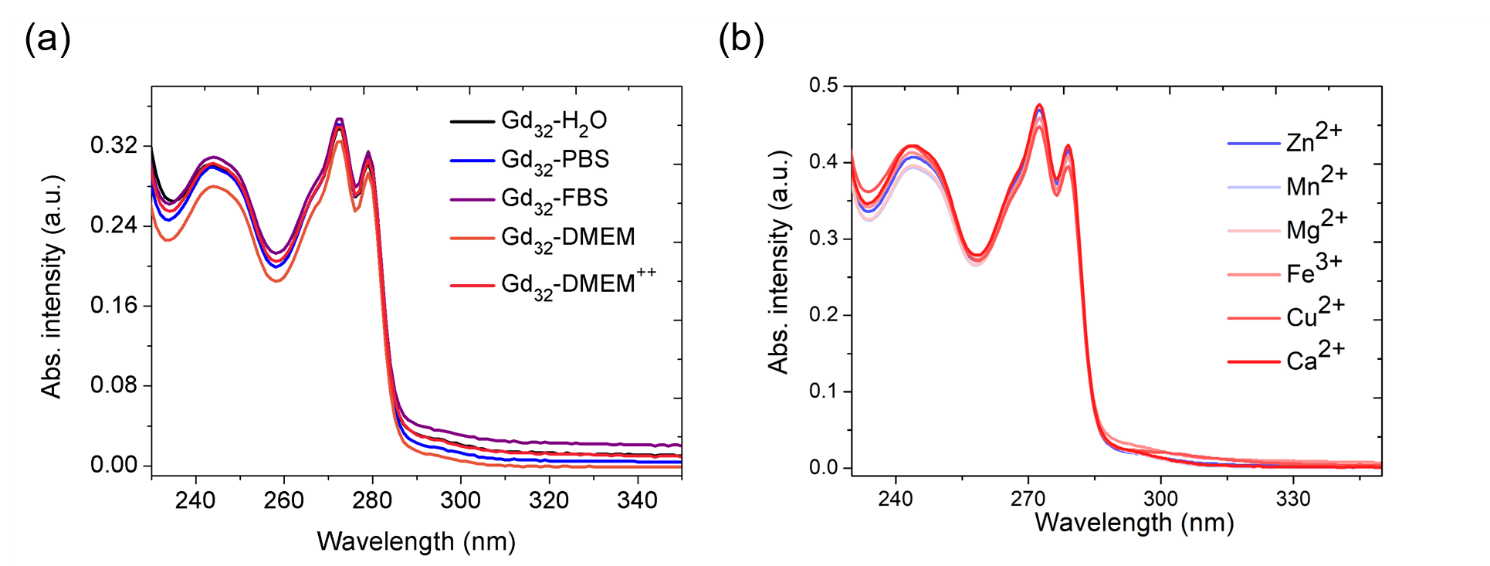


# Figure S17. UV-Vis absorption spectra of Gd_32_ in PBS, serum (FBS), cell culture medium (DMEM and DMEM^++^, DMEM^++^ denotes DMEM medium containing penicillin and streptomycin) and PBS solution containing endogenous metal ions (Ca^2+^, Mg^2+^, Fe^3+^, Zn^2+^, *etc.*).


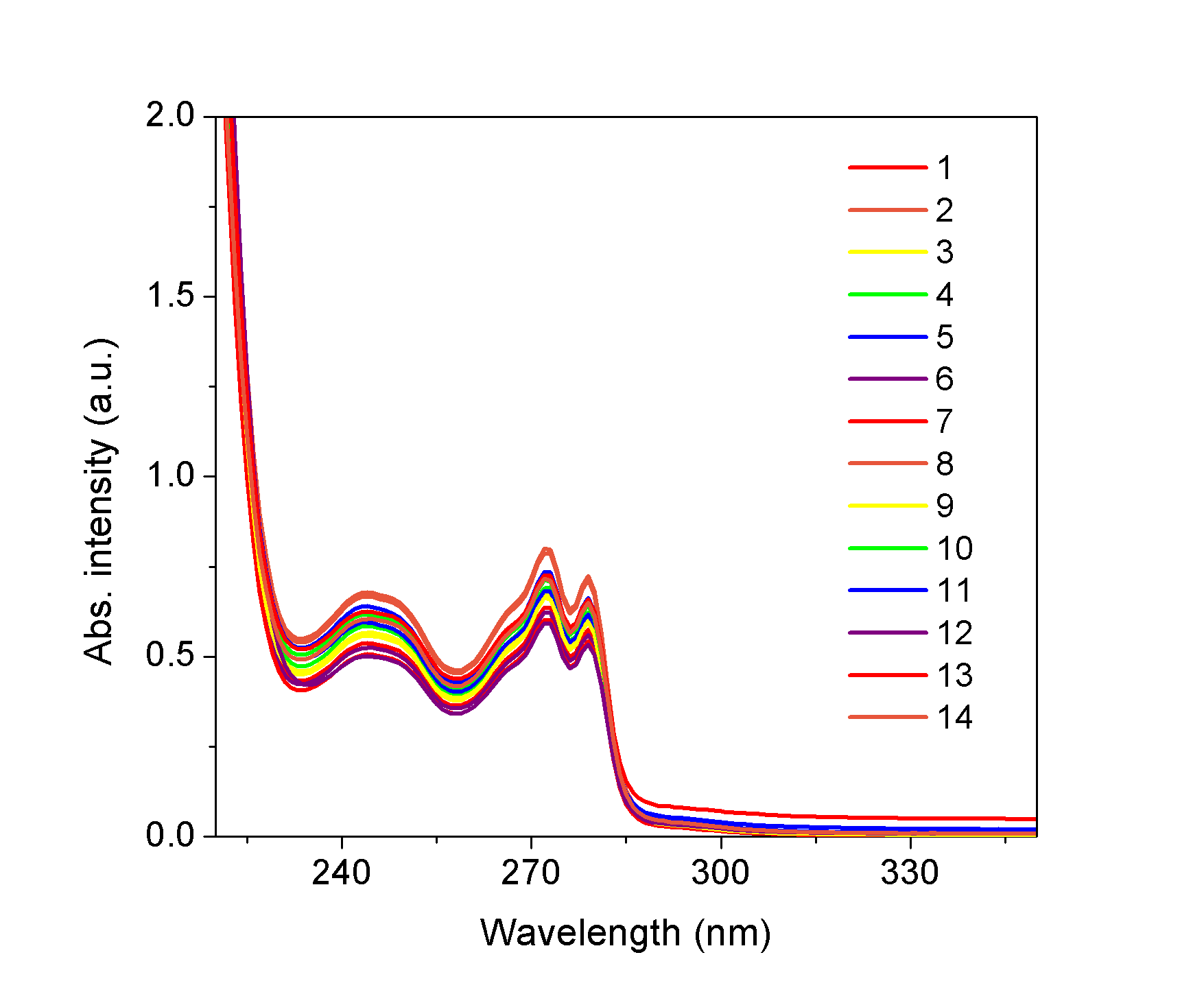


# Figure S18. UV-Vis absorption spectra of Gd_32_ in aqueous solutions of different pH (1-14).


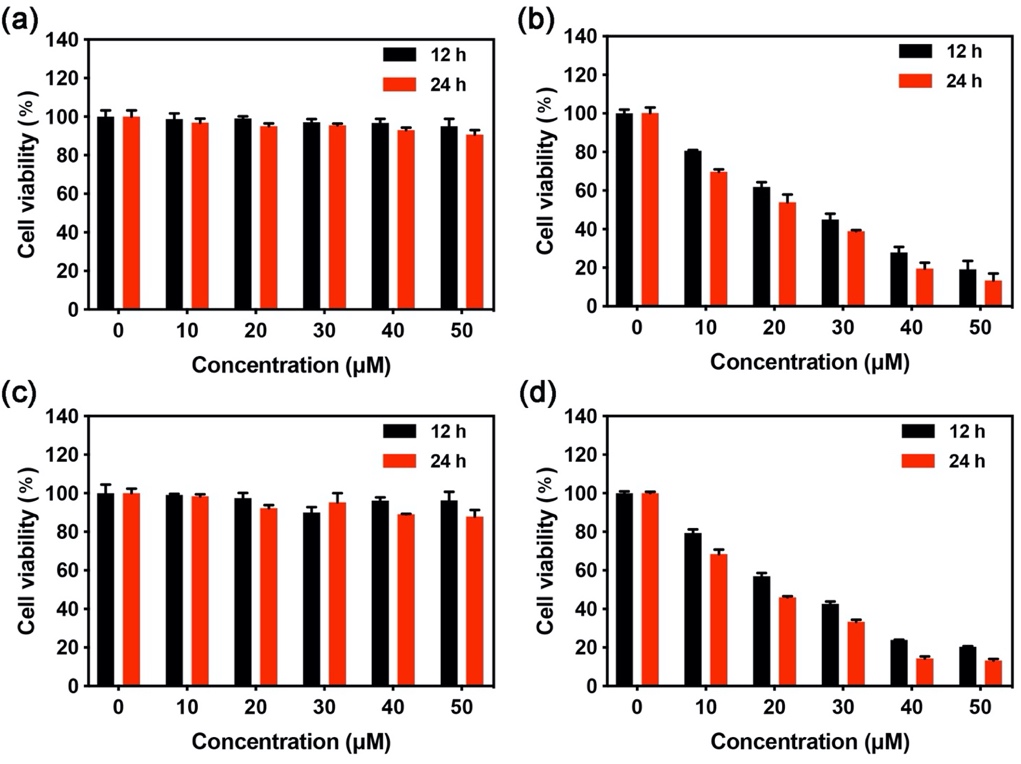


# Figure S19. Cell viability for HUVEC and 4T1 cells after incubation with cluster Gd_32_ (a, c) and cisplatin (b, d) for 12 h and 24 h, respectively.

**Toxicity Analysis of Spherical Cluster Gd_32_.** In order to explore the toxicity of globular **Gd_32_** clusters to different types of cells, we used the MTT method to test the antiproliferative effects of **Gd_32_** at different concentrations on HUVECs and 4T1 cells. It is worth noting that the cluster **Gd_32_** aqueous solution with a concentration range of 0-50 μM did not show obvious toxicity after incubating with HUVECs and 4T1 cells for 12 h. As the incubation time increased to 24 h, aqueous solution **Gd_32_** was still nontoxic to HUVECs and 4T1 cells. Therefore, a comparison with cisplatin shows that the globular cluster **Gd_32_** exhibits negligible cytotoxicity (Figure S19-S20). Immediately thereafter, we conducted a hemolysis test on **Gd_32_**. As the concentration of **Gd_32_** increased from 0 μM to 40 μM, no significant hemolysis was observed, which indicates that **Gd_32_** has no obvious damage to the red blood cell wall and can be administered by intravenous injection (Figures S20b and S21). In addition, **Gd_32_** was injected intravenously into BALB/c mice at a dose of 10 mg/kg, and the weight changes of the mice were monitored for a week. Compared with the control group that was not administered **Gd_32_**, the two groups of mice had similar growth rates in regard to their weights (Figure S20c). Similarly, routine blood tests were performed for the healthy mice and mice that were injected with **Gd_32_** and the results showed that there was no significant difference between the two groups (Figure S20d). Finally, the histological analysis involving the important organs (heart, liver, spleen, lung and kidney) of the mice one week after the injection of **Gd_32_** showed that the **Gd_32_** did not cause significant tissue toxicity (Figure S20e). Overall, the abovementioned many results indicate that **Gd_32_** shows negligible toxicity at the cell and animal levels and is very suitable for the next step of in vivo MRI exploration.


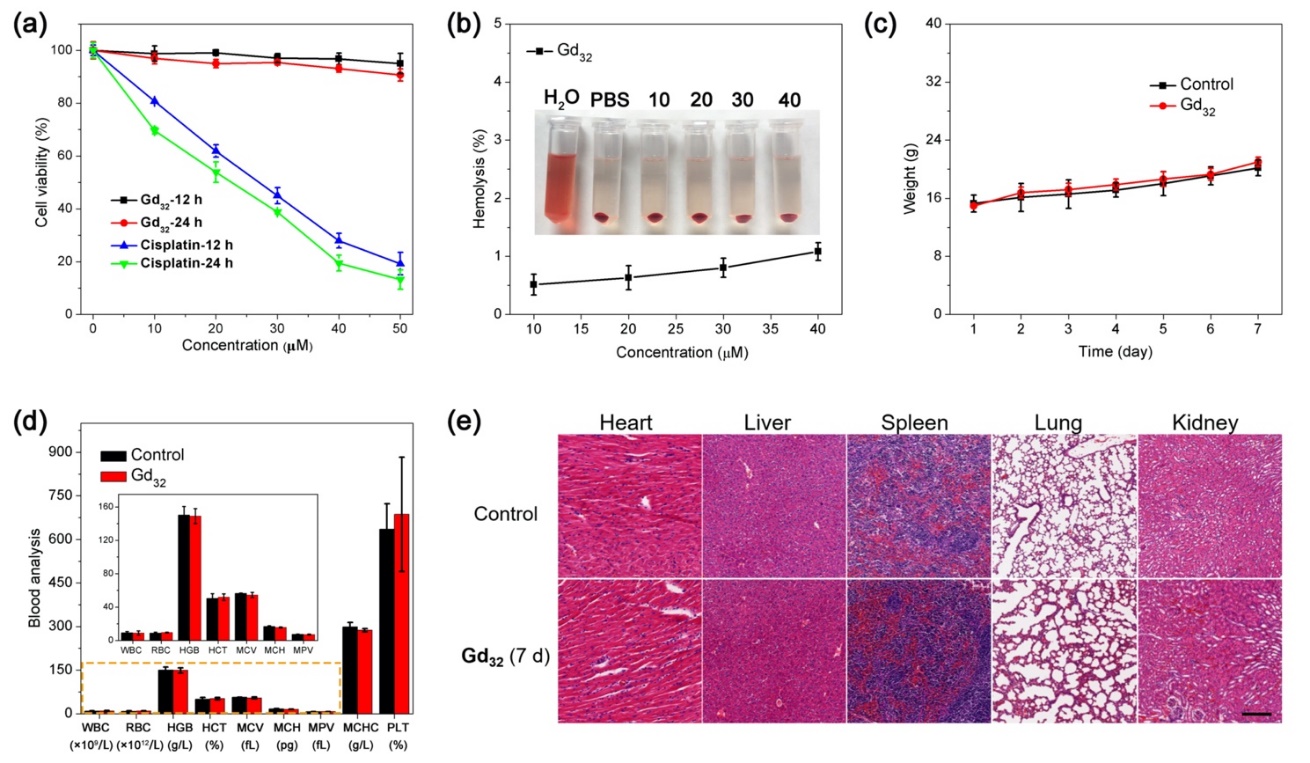


# Figure S20. (a) Cell viability of HUVECs after incubation with Gd_32_ and cisplatin for 12 h and 24 h. (b) Red blood cells incubated with various concentrations of Gd_32_ for 12 h (PBS and pure water were employed as the negative and positive controls, respectively). (c) Body weight changes of mice in the intravenously injected Gd_32_ at a dose of 10 mg/kg and mice in the noninjected group (blank) within one week. (d) Routine blood tests of healthy mice and mice injected with Gd_32_. (e) Histopathological examination of the main organs of mice injected with PBS (control) and Gd_32_, respectively (Scale bar: 100 µm).


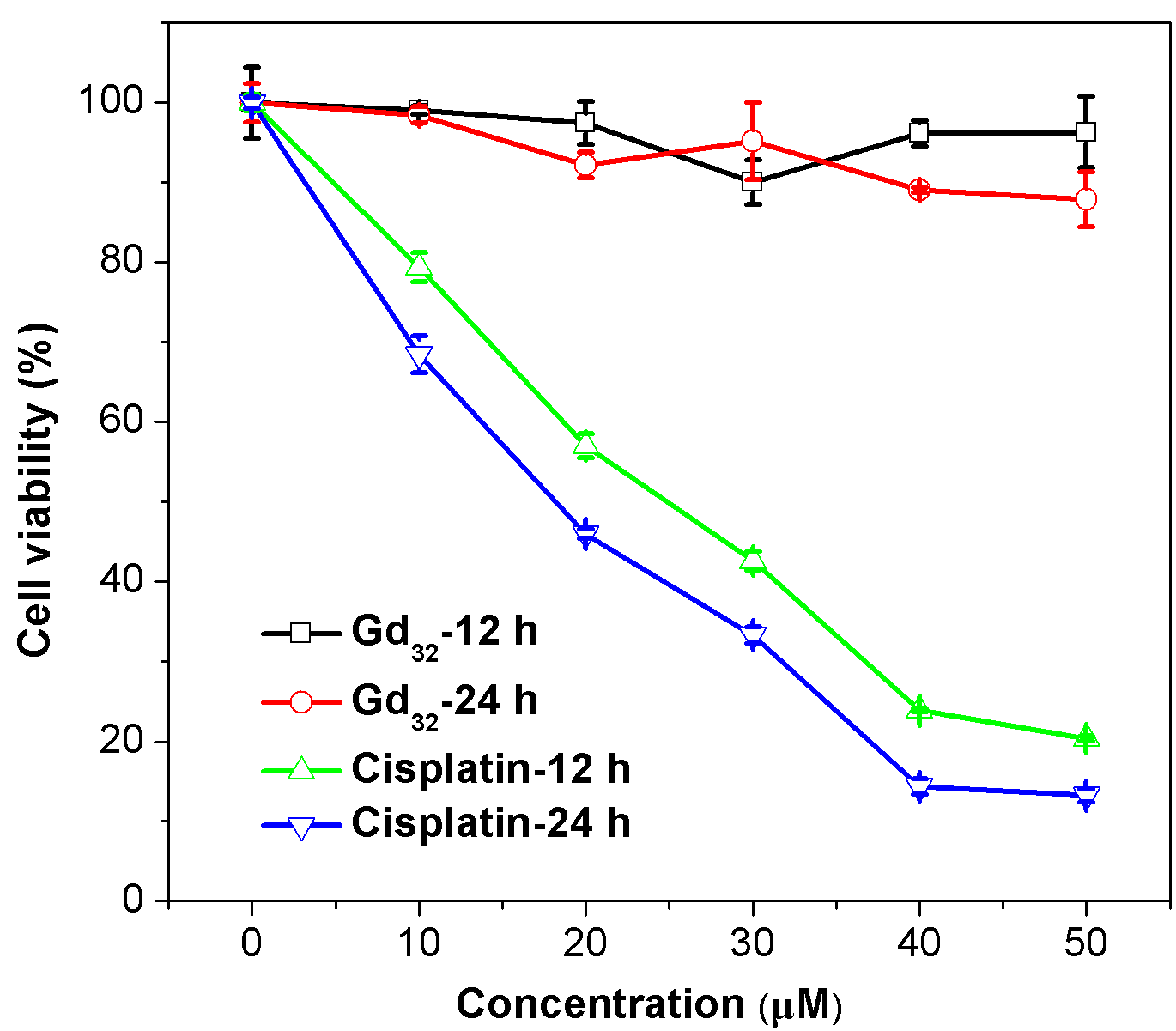


# Figure S21. Comparison of the cytotoxicity of cluster Gd_32_ and cisplatin after co-incubation with 4T1 cells for 12 h and 24 h, respectively.


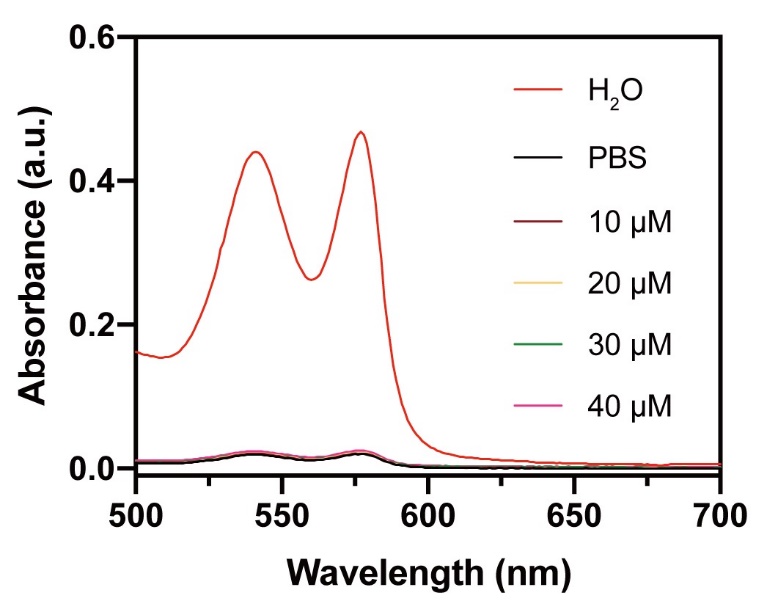


# Figure S22. Ultraviolet-visible (UV-Vis) absorption spectrum of hemolysis experiments with cluster Gd_32_ of different concentrations.


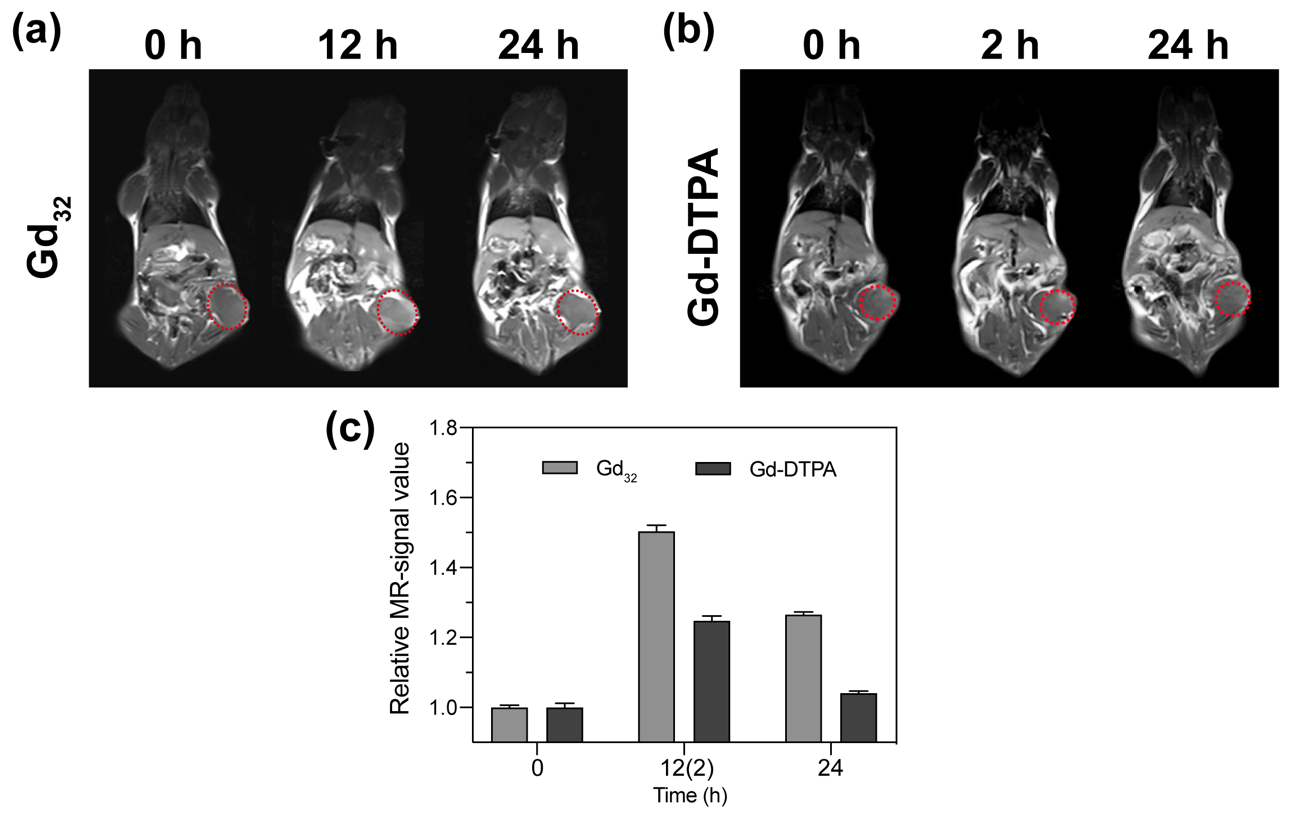


# Figure S23. MR-imaging in vivo at 3 T: After injecting cluster Gd_32_ (a) and commercial contrast agents Gd-DTPA (b) into BALB/c mice carrying the 4T1 tumor model through the tail vein, the MRI images of the mice at different time points, the circular frame is the tumor site; (c) The relative MR-signal value of tumors at different time points of the mice injected with cluster Gd_32_ and commercial contrast agents Gd-DTPA, respectively.

# Reference

1. Sheldrick GM. *Acta Crystallogr., Sect. C: Struct. Chem.* 2015; **71**: 3–8.
2. Spek, AL. PLATON SQUEEZE: a tool for the calculation of the disordered solvent contribution to the calculated structure factors. *Acta Cryst.* 2015; **C71**: 9–18.
3. Outten CE and O’Halloran TV. Femtomolar Sensitivity of Metalloregulatory Proteins Controlling Zinc Homeostasis. *Science* 2001; **292**: 2488–92.
4. Alvarez S, Alemany P and Casanova D *et al.* Shape maps and polyhedral interconversion paths in transition metal chemistry. *Coord Chem Rev* 2005; **249**: 1693−708.
5. Qin L, Zhou GJ and Yu YZ *et al.* Topological Self-Assembly of Highly Symmetric Lanthanide Clusters: A Magnetic Study of Exchange-Coupling “Fingerprints” in Giant Gadolinium(III) Cages. *J Am Chem Soc* 2017; **139**: 16405–11.
6. He Q, Huang H and Zheng XY *et al.* Polymer-Encapsulated Lanthanide-Containing Clusters as Platforms for Fabricating Magnetic Soft Materials. *ACS Appl. Mater. Interfaces* 2018; **10**: 16947–51.
7. Ji C, Liu S and Su K *et al.* Pyrogallol[4]Arene Coordination Nanocapsule Micelle as Bioinspired Water Reduction Catalyst. *ACS Mater. Lett.* 2021; **3**: 1315–20.
8. Luo ZR, Wang HL and Zhu ZH *et al.* Assembly of Dy_60_ and Dy_30_ Cage-Shaped Nanoclusters. *Commun Chem* 2020; ***3***(1): 30. <https://doi.org/10.1038/s42004-020-0276-3>.
9. Zheng H, Du MH and Lin SC *et al.* Assembly of a Wheel-Like Eu_24_Ti_8_ Cluster under the Guidance of High-Resolution Electrospray Ionization Mass Spectrometry. *Angew Chem Int Ed* 2018; **57**: 10976–79.
10. Wang HL, Ma XF and Peng JM *et al.* Tracking the Stepwise Formation of the Dysprosium Cluster (Dy_10_) with Multiple Relaxation Behavior. *Inorg Chem* 2019; **58**: 9169–74.
11. Huang W, Chen W and Bai Q *et al.* Anion‐Guided Stepwise Assembly of High‐Nuclearity Lanthanide Hydroxide Clusters. *Angew. Chemie Int. Ed.* 2022; **61**: e202205385.
12. Wang Z, He L and Liu B *et al.* Coordination-Assembled Water-Soluble Anionic Lanthanide Organic Polyhedra for Luminescent Labeling and Magnetic Resonance Imaging. *J Am Chem Soc* 2020; **142**: 16409–19.
13. Wahsner J, Gale EM and Rodríguez-Rodríguez A *et al.* Chemistry of MRI Contrast Agents: Current Challenges and New Frontiers. *Chem Rev* 2019; **119**: 957–057.
14. Merbach A, Helm L and Tóth, É. *The Chemistry of Contrast Agents in Medical Magnetic Resonance Imaging*; Merbach, A., Helm, L., Tóth, É., Eds.; John Wiley & Sons, Ltd: Chichester, UK, 2013. <https://doi.org/10.1002/9781118503652>.
15. Li XZ, Tian CB and Sun QF. Coordination-Directed Self-Assembly of Functional Polynuclear Lanthanide Supramolecular Architectures. *Chem Rev* 2022; **122**: 6374–458.
16. Ananta JS, Godin B and Sethi R *et al.* Geometrical Confinement of Gadolinium-Based Contrast Agents in Nanoporous Particles Enhances T_1_ Contrast. *Nat. Nanotechnol.* 2010; **5**: 815–21.
17. Lauffer RB, Paramagnetic Metal Complexes as Water Proton Relaxation Agents for NMR Imaging: Theory and Design. *Chem Rev* 1987; **87**: 901–27.
18. Marangoni VS, Neumann O and Henderson L *et al.* Enhancing T_1_ Magnetic Resonance Imaging Contrast with Internalized Gadolinium(III) in a Multilayer Nanoparticle. *Proc Natl Acad Sci* 2017; **114**: 6960–5.
19. Powell DH, Dhubhghaill OMN and Pubanz D *et al.* Structural and Dynamic Parameters Obtained from ^17^O NMR, EPR, and NMRD Studies of Monomeric and Dimeric Gd^3+^ Complexes of Interest in Magnetic Resonance Imaging: An Integrated and Theoretically Self-Consistent Approach 1. *J Am Chem Soc* 1996; **118**: 9333–46.
20. Rodríguez-Rodríguez A, Esteban-Gómez D and Blas A *et al.* Lanthanide(III) Complexes with Ligands Derived from a Cyclen Framework Containing Pyridinecarboxylate Pendants. The Effect of Steric Hindrance on the Hydration Number. *Inorg Chem* 2012; **51**: 2509–21.
21. Webber BC, Payne KM and Rust LN *et al.* Analysis of the Relaxometric Properties of Extremely Rapidly Exchanging Gd^3+^ Chelates: Lessons from a Comparison of Four Isomeric Chelates. *Inorg Chem* 2020; **59**: 9037–46.
